# Trolox, Ferulic, Sinapic, and Cinnamic Acid Derivatives of Proline and GABA with Antioxidant and/or Anti-Inflammatory Properties

**DOI:** 10.3390/molecules29163763

**Published:** 2024-08-08

**Authors:** Georgios Papagiouvannis, Panagiotis Theodosis-Nobelos, Eleni A. Rekka

**Affiliations:** 1 Department of Pharmacy, School of Health Sciences, Frederick University, Nicosia 1036, Cyprus; hsc.pag@frederick.ac.cy (G.P.); hsc.np@frederick.ac.cy (P.T.-N.); 2 Department of Pharmaceutical Chemistry, School of Pharmacy, Aristotle University of Thessaloniki, 54124 Thessaloniki, Greece

**Keywords:** neurodegeneration, oxidative stress, inflammation, multi-targeting compounds, acetylcholinesterase inhibitors, proline, GABA

## Abstract

Degenerative conditions, such as neurodegenerative disorders (Alzheimer’s disease (AD), Parkinson’s disease (PD)) and cardiovascular diseases, are complex, multifactorial disorders whose pathophysiology has not been fully elucidated yet. As a result, the available treatment options cannot eliminate these diseases radically, but only alleviate the symptoms. Both inflammatory processes and oxidation are key factors in the development and evolution of neurodegeneration, while acetylcholinesterase inhibitors are the most used therapeutic options against AD. In this work, following the multi-targeting compound approach, we designed and synthesized a series of proline and gamma-aminobutyric acid (GABA) amides with various acidic moieties that possess an antioxidant and/or anti-inflammatory potency. Proline is the pharmacophore of nootropic drugs (e.g., piracetam) used for memory improvement, while GABA is the main inhibitory neurotransmitter in the central nervous system. The designed molecules were subjected to a preliminary screening of their bioactivity in antioxidant and anti-inflammatory assays, as well as against acetylcholinesterase. Most of the synthesized compounds could inhibit lipid peroxidation (IC_50_ as low as 8 μΜ) and oxidative protein glycation (inhibition of up to 48%) and reduce the 2,2-diphenyl-1-picrylhydrazyl free radical (DPPH). In addition, all of the compounds were moderate inhibitors of lipoxygenase (LOX) (up to 46% at 100 μΜ) and could decrease carrageenan-induced paw edema in rats by up to 55%. Finally, some of the compounds were moderate acetylcholinesterase inhibitors (IC_50_ as low as 219 μΜ). The results confirmed the design rationale, indicating that the compounds could be further optimized as multi-targeting molecules directed against degenerative conditions.

## 1. Introduction

The term “neurodegenerative disorders” includes a variety of diseases characterized mainly by gradual neuron loss and dysfunction. As the life expectancy increases, so does the possibility of neurodegeneration development, which makes the discovery of new, more effective therapeutic agents necessary [1]. Alzheimer’s disease (AD) is the most common neurodegenerative disorder, accounting for approximately 70% of dementia cases worldwide [2]. Its main symptoms include memory loss, cognitive impairment, and behavioral disorders and its pathophysiology is characterized by tau protein hyperphosphorylation and amyloid precursor protein (APP) decomposition, leading to the formation of neurofibrillary tangles (NFTs) and Aβ amyloid peptide aggregation, respectively [3]. Although significant progress has been made in understanding AD pathophysiology and pathobiochemistry, the mechanisms that contribute to the initiation of neurodegeneration have not been fully elucidated. As a result, there are no available treatment options for eliminating AD radically, but only to alleviate the symptoms.

Oxidative stress has been found to play a key role in the progression of neurodegeneration. Since the brain consumes more than 20% of the respirated oxygen, oxidative stress is highly established in the central nervous system (CNS) [4]. Amyloid peptide aggregation and tau protein hyperphosphorylation are induced by extensive oxidative damage and advanced glycation end-products (AGEs), whereas mitochondrial dysfunction has been found to be of great significance for AD pathogenesis, as it is closely related to cell death and neurodegeneration [5,6,7]. Furthermore, the excitotoxicity produced by increased N-methyl-D-aspartate (NMDA) signaling enhances reactive oxygen species (ROS) formation, resulting in cell malfunction and neurodegeneration [8].

The neuroinflammation induced by microglial cells and astrocyte activation is another key factor in AD pathogenesis, as it is accountable for structural and functional changes in CNS cells, leading to apoptosis and neural loss [9]. Prostaglandins, especially PGE2, inhibit amyloid phagocytosis, thus enhancing the neurotoxicity of microglia, and the lipoxygenase (LOX) activity is closely associated with amyloid plaques and NFT formation [10,11]. Various chronic diseases may have different etiologies; however, prolonged oxidative stress is a common characteristic of these diseases. In aging and age-related conditions, oxidative stress is interrelated with inflammation in a two-way communication. Oxidative stress can induce and aggravate inflammation and vice versa, establishing a vicious cycle [12].

According to the cholinergic hypothesis, a decrease in the cholinergic neurotransmission caused by cholinergic neuron depletion contributes to memory loss and cognitive impairment during AD [13]. Since the acetylcholine (Ach) levels are lowered in the brains of patients with AD, acetylcholinesterase (AchE) inhibitors, which increase the Ach concentration, have been proven to be useful agents for the amelioration of AD symptoms [14]. Nevertheless, AchE inhibitors have failed to treat AD effectively, pointing out that the cholinergic hypothesis needs to be reconsidered and further studies about the relations of AchE with amyloid plaques, NFTs, and other factors taking part in AD pathogenesis have to be carried out [15].

By taking into account all the above-mentioned information and considering the multi-targeting compound approach [16], we designed and synthesized a series of novel compounds by the amidation of proline and GABA with various carboxylic acids. A proline ring is the main pharmacophore of nootropic drugs (e.g., piracetam) used for memory improvement [17], whereas GABA is the main inhibitory neurotransmitter in the CNS [18]. The carboxylic acids used for the synthesis of our derivatives included ((*E*)-3-methoxy-4-hydroxyphenyl)acrylic acid (ferulic acid) for compound **1c** and ((*E*)-3,5-dimethoxy-4-hydroxyphenyl)acrylic acid (sinapic acid) for compound **2c**, which exert antioxidant and neuroprotective activity in cells [19,20]; (*E*)-3,4-dimethoxy-phenyl)acrylic acid for compound **3c** and cinnamic acid for compound **4c**, which possess an anti-inflammatory potency due to their cinnamic residue [21]; and Trolox (6-hydroxy-2,5,7,8-tetramethylchroman-2-carboxylic acid) for compound **5c**, which is a well-known water-soluble derivative of tocopherol (vitamin E) with an antioxidant capacity [22]. The bioactivity of these carboxylic acids and some of their derivatives has been extensively reported in the literature. More specifically, ferulic acid and various ferulic derivatives have demonstrated DPPH-reducing activity and a lipid oxidation inhibitory capacity [23]. In addition, Trolox amides have been found to act as radical scavengers (very good DPPH-reducing activity) and as moderate acetylcholinesterase inhibitors [24]. Sinapic ester derivatives have been proven to exert lipid peroxidation inhibitory activity [25], while plant extracts containing phenolic acid derivatives have been proven to inhibit lipid peroxidation and albumin oxidation, as well as reduce DPPH radicals [26,27]. Based on these results, we suggest that compounds with antioxidant and/or anti-inflammatory characteristics could be of great interest for the effective treatment of degenerative conditions, such as neurodegenerative disorders and cardiovascular diseases.

**Figure 1:**
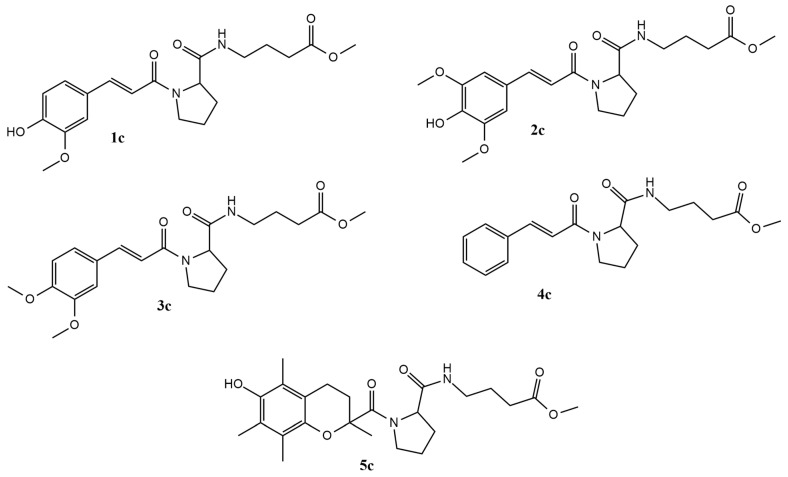
Depiction of the synthesized molecules.

All the compounds (Figure 1) were tested as antioxidants. In particular, their activity against rat hepatic microsomal membrane lipid peroxidation, their interaction ability with the stable free radical 2,2-diphenyl-1-picrylhydrazyl (DPPH), and their potency against fructose-induced protein glycation were evaluated. Moreover, they were examined for their inhibition against AchE, while their anti-inflammatory properties were evaluated by the LOX inhibition capacity and their effect on rat paw edema induced by carrageenan.

## 2. Results

### 2.1. Synthesis

**Scheme 1:**
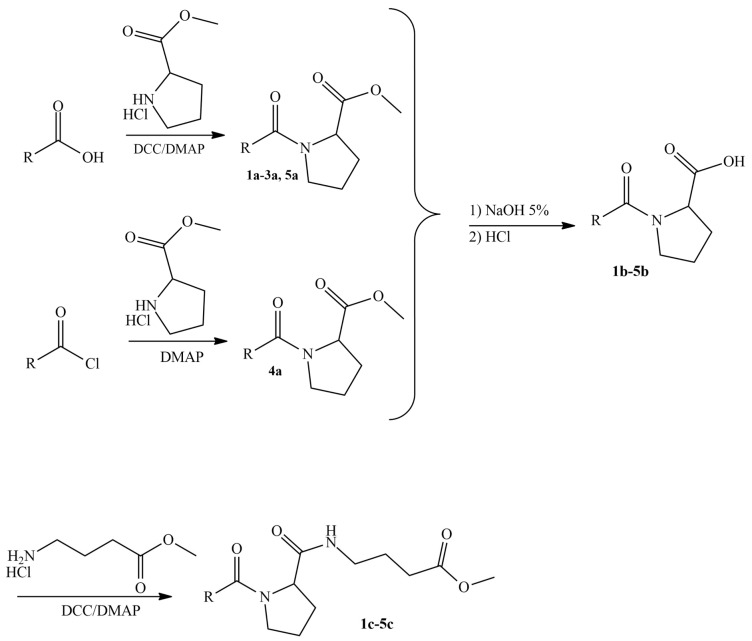
Synthetic procedure of compounds **1c**–**5c**.

As shown in Scheme 1, the synthetic process was carried out in three steps. In the first step, the respective carboxylic acids were amidated with proline methyl ester and the produced intermediates (compounds **1a**–**5a**) were hydrolyzed in the second step. Finally, the carboxylic acids obtained from the second step (compounds **1b**–**5b**) were amidated with GABA methyl ester and the final molecules (compounds **1c**–**5c**) were isolated in yields of up to 78%.

### 2.2. Antioxidant Activity

The synthesized compounds were tested for their inhibitory activity against the lipid peroxidation of rat hepatic microsomal membranes. The IC_50_ values of the compounds after 45 min of incubation are shown in Table 1.

**Figure 2:**
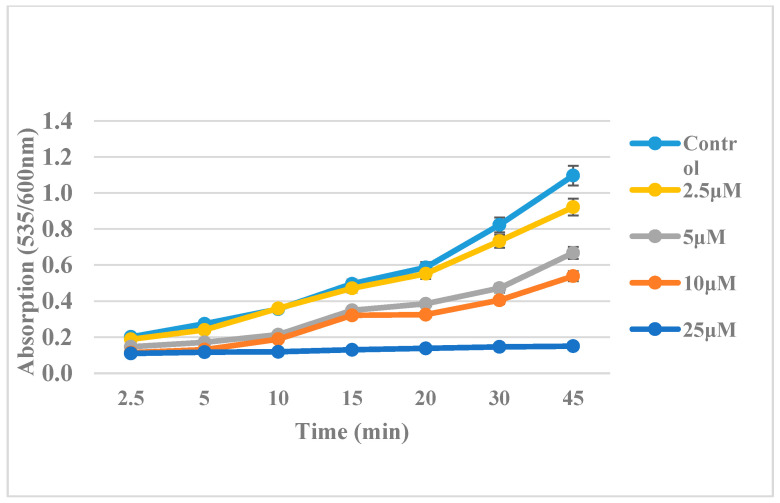
Effect of various concentrations of compound **2** on time course of lipid peroxidation.

The time course of lipid peroxidation, as affected by various concentrations of the most active compound (**5c**), is shown in Figure 2.

The antioxidant capacity of our compounds was also estimated by their reducing ability for DPPH free radicals (Table 2).

**Figure 3:**
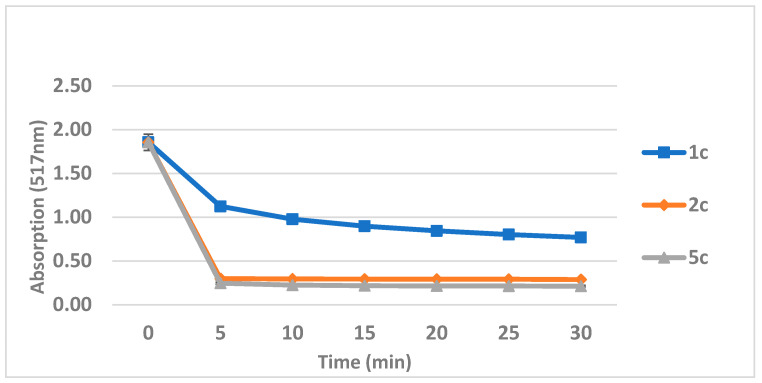
DPPH interaction with compounds **1c**, **2c**, and **5c** (concentration of 200 μΜ) as a function of time.

The time course of the DPPH reaction with the compounds **1c**, **2c**, and **5c** is shown in Figure 3.

Furthermore, the antioxidant potency of the designed compounds was estimated by their inhibitory activity against copper-induced oxidative protein glycation. The compounds were dissolved in water, but a small amount of dimethylsulfoxide (DMSO) was added in some cases due to low water solubility. DMSO was found not to affect the glycation process. The inhibitory activity against the protein glycation of compounds **1**–**6** is shown in Table 3.

### 2.3. Acetylcholinesterase Inhibition

The IC_50_ values, as well as the molecular volumes of compounds **1c**–**5c**, are shown in Table 4.

### 2.4. Anti-Inflammatory Properties

The inhibitory activity of the designed compounds towards soybean lipoxygenase, as well as their clgoP values, are demonstrated in Table 5.

Furthermore, the anti-inflammatory efficacy of the compounds was estimated in vivo by their ability to reduce carrageenan-induced edema in rat paws. The effect of the compounds on paw edema, as well as the respective activity of ibuprofen and naproxen (non-steroidal anti-inflammatory drugs used as references), are shown in Table 6.

## 3. Discussion

### 3.1. Chemistry

The synthesis of our derivatives was carried out in three steps. In the first step, the respective carboxylic acid was amidated with methyl pyrrolidine-2 carboxylate hydrochloride (proline methyl ester hydrochloride) using dicyclohexylcarbodiimide (DCC) as a coupling agent and N,N-dimethyl-aminopyridine (DMAP). For the synthesis of compound **4a**, the commercially available cinnamyl chloride was used as the starting material. The reactions were carried out in dichloromethane, using drops of dimethylformamide (DMF) due to the low solubility of the starting carboxylic acid. The expected intermediates were isolated with flash column chromatography in yields of up to 90%. In the second step, ester derivatives **1a**–**5a** were hydrolyzed using a 5% aqueous NaOH solution and 1,4-dioxane as a solvent. The expected carboxylic acids were extracted with ethyl acetate from the aqueous phase after acidification and were obtained in yields of 86–97%. Finally, in the third step, compounds **1b**–**5b** were amidated with methyl-4-aminobutanoate hydrochloride (GABA methyl ester hydrochloride) using DCC as a coupling agent and DMAP. The reactions were performed in dichloromethane and the expected products were purified and received with flash column chromatography in moderate yields, with the maximum reaching up to 78%.

### 3.2. Antioxidant Activity

As expected, compounds **3c** and **4c** could not inhibit lipid peroxidation, due to the lack of antioxidant structural characteristics. A group with an easily abstracted hydrogen atom is usually present in antioxidant compounds such as phenols. The phenoxyl radical formed was stabilized by conjugation with the phenyl ring. Compounds **3c** and **4c** did not have any easily abstracted hydrogen atoms. Compound **5c** (Trolox derivative) was the most active, demonstrating an IC_50_ value of 8 μΜ, about three times more active than Trolox. Trolox is a vitamin E analogue with a strong antioxidant potency; therefore, its derivatives are expected to possess a high level of antioxidant activity. The higher antioxidant activity of compound **5c** compared to Trolox can be attributed to its higher lipophilicity, which leads to a more effective approach to the membrane lipids. Compounds **1c** and **2c** had a lower inhibitory activity against lipid peroxidation, due to both the lower lipophilicity and low electron-donating effect of methoxy substituents, which could not stabilize the phenoxyl radical effectively. Comparing the activity of **1c** and **2c**, the mesomeric effect of the monomethoxy substituent in compound **1c** led to a less stable phenoxyl radical than the dimethoxy substituents, and as a result, the sinapic derivative **2c** was a more potent lipid peroxidation inhibitor than the ferulic analogue **1c**. The effect of lipophilicity in the inhibitory activity against lipid peroxidation has been confirmed by previous studies in our lab, in which ferulic, sinapic, and Trolox analogues with higher clogP values had a stronger efficacy as lipid peroxidation inhibitors [28].

Moreover, compound **5c** was the most active in reducing DPPH. It had a similar activity to that of Trolox at 200 μΜ and 100 μΜ (equal to and half of the DPPH concentration, respectively) and a slightly higher activity at 50 μΜ and 25 μΜ. Compound **2c** was highly active at 200 μΜ, but not in lower concentrations, while compound **1c** had a lower reducing capacity, even at 200 μΜ. Compounds **2c** and **5c** could interact rapidly with DPPH since their reaction was completed in the first five minutes, while the interaction between **1c** and DPPH was somewhat slower, being completed in about 15 min. The results aligned with the lipid peroxidation inhibition results, pointing out that **5c** is a stronger antioxidant agent than compounds **1c** and **2c**. Since DPPH is a lipophilic free radical, compounds with a high lipophilicity can approach it effectively, thus being able to present a greater DPPH-reducing activity. Indeed, derivatives with a high lipophilicity synthesized previously by us exerted a high level of reducing activity against DPPH [28,29]. Compounds **3c** and **4c**, with no antioxidant moieties, could not interact with DPPH at all.

Finally, the antioxidant capacity of our molecules was estimated by their inhibitory activity against oxidative protein glycation induced by copper ions. AGEs demonstrate neurotoxic effects, as they increase the APP levels and induce its degradation by beta-secretase. Moreover, they promote apoptosis-related gene expression, leading to neural loss [30]. Despite demonstrating a high antioxidant potency as a lipid peroxidation inhibitor and DPPH reducing agent, compound **5c** did not show as high a protein glycation inhibitory activity level as compounds **1c** and **2c**. Its low water solubility combined with a more rigid structure may not have allowed the molecule to approach bovine albumin effectively, and thus prevented it from inhibiting oxidative albumin glycation. The higher anti-glycation activity of compounds **1c** and **2c** can be attributed to their acidic properties (phenolic derivatives), since albumin has a great number of basic amino acids, which can bind with acidic molecules [31]. Furthermore, an increased ability to form hydrogen bonds due to their multiple hydrogen acceptor groups, allowing a more effective approach between the molecules and the albumin, resulted in higher inhibitory activity against glycation [32]. Compounds **3c** and **4c** did not exert any inhibition against protein glycation, due to the absence of acidic substituents and antioxidant structural characteristics.

### 3.3. Acetylcholinesterase INHIBITION

**Figure 4:**
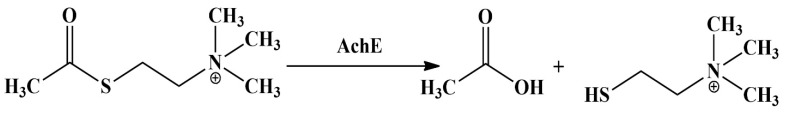
Acetylthiocholine breakdown.

Acetylcholinesterase (AchE) is the main pharmacological target of most anti-Alzheimer drugs, since cholinergic neuron loss has been closely related to memory loss and cognitive impairment [33]. The anti-AchE activity of the designed compounds was evaluated by their inhibition against acetylthiocholine hydrolysis, induced by AchE and derived from rat brain homogenate (Figure 4).

Compounds **1c**–**3c** exerted moderate inhibitory activity against AchE, which can be attributed to the hydroxy- and methoxy-groups on the benzene ring that may have improved the effective bondage between the compound and the enzyme. No inhibition was observed in the presence of higher concentrations of acetylthiocholine, indicating that the designed compounds could act as competitive AchE inhibitors. The ferulic analogue **1c** was slightly more active than **2c** and **3c**, a result consistent with previous results that demonstrated the anti-AchE activity of various ferulic analogues [34]. The lower efficacy of compounds **2c** and **3c** may be attributable to their larger molecular volume, which did not allow them to approach the active site of AchE effectively, despite having aromatic substituents contributing to the binding to the enzyme. Compound **5c** had an even larger molecular volume, since the chroman ring is a bulky moiety; therefore, it was unable to interact with the active center of AchE and exerted no inhibitory activity. Finally, compound **4c**, despite its low molecular volume, did not bind to the enzyme due to the absence of aromatic substituents.

### 3.4. Anti-Inflammatory Activity

Lipoxygenases catalyze arachidonic acid metabolism using molecular oxygen for the peroxidation of their substrate [35]. Their activity increases with age and is highly associated with AD pathogenesis, promoting amyloid plaque deposition and NFT formation [36,37].

All the compounds demonstrated moderate inhibitory activity against LOX (up to 40% at 100 μΜ), with compounds **2c** and **4c** being the most potent inhibitors. Despite its strong antioxidant potency, both as a lipid peroxidation inhibitor and as a DPPH radical scavenger, compound **5c** was the least active LOX inhibitor. It can be assumed that the inhibitory activity is not attributable to the ability of the compounds to reduce free radicals produced by LOX, but to their ability to bind to the enzyme and prevent substrate binding. No inhibitory activity was observed when higher substrate (linoleic acid) concentrations were added, indicating that the designed compounds acted as weak competitive LOX inhibitors.

Finally, our compounds were tested in vivo for their ability to reduce rat paw edema induced by carrageenan. Carrageenan-induced inflammation causes histamine and serotine release (90 min after the administration) and continues with kinins secretion (2.5 h after the injection). During the last phase (more than 2.5 h after administration), prostaglandins are produced, and pro-inflammatory cytokines are released [38]. In our protocol, the inflammatory process was estimated 3.5 h after the carrageenan administration. Compounds **1c**, **2c**, and **5c** exerted a high activity, which is consistent with their antioxidant potential and may be of great importance for their anti-inflammatory potency, as we have previously reported [39]. The anti-inflammatory properties of the respective carboxylic acids have been previously reported; therefore, these derivatives are also expected to possess anti-inflammatory activity [20,40]. Compound **3c** demonstrated the strongest reducing activity against edema (55%), although it had no antioxidant efficacy. This can be explained by the fact that not only oxidative, but also various other biochemical procedures are involved in inflammatory processes. Taking into account the low LOX inhibitory activity of the designed compounds and the marked reduction in edema in vivo, we hypothesize that many other factors may be related to the inflammatory responses induced by carrageenan. All the rats that received the test compounds appeared normal, macroscopically and by autopsy, at the end of the procedure.

## 4. Materials and Methods

### 4.1. General

All the commercially available chemicals were purchased from Merck (Kenilworth, NJ, USA) or Sigma (St. Lewis, MO, USA). The NMR spectra (^1^H-NMR and ^13^C-NMR) were taken using an AGILENT DD2-500 MHz spectrometer. The IR spectra were recorded on a Perkin Elmer Spectrum BX FT-IR spectrometer (Waltham, MA, USA). The MS method would have been very useful for an additional confirmation of the structure and purity of the synthesized compounds, but unfortunately, we currently do not have access to such an apparatus. An MEL-TEMPII apparatus from Sigma-Aldrich was used for the determination of the melting points. The microanalyses were carried out on a Perkin-Elmer 2400 CHN elemental analyzer (Waltham, MA, USA). The progress of reactions was monitored using thin-layer chromatography (TLC silica gel 60 F24 aluminum sheets, Merck) and the spots were visualized under ultraviolet light.

### 4.2. Synthesis

#### 4.2.1. Synthesis of Compounds **1a**–**3a** and **5a**

The corresponding carboxylic acid (3 mmol) was dissolved in dry dichloromethane using a small amount of DMF (up to 0.5 mL) to increase the solubility. Then, methyl pyrrolidine-2-carboxylate hydrochloride (3.6 mmol) and N,N-dimethylaminopyridine (DMAP, 3.6 mmol), and, subsequently (15 min afterwards), N,N dicyclohexylcarbodiimide (DCC, 3.6 mmol), were added, and the mixture was stirred at ambient temperature overnight. The resulting mixture was purified with filtration and washed with HCl (5%), NaHCO_3_ (5%), and a saturated NaCl solution, and then dried over Na_2_SO_4_. Finally, the solvent was distilled off under a low pressure and the final product was purified via flash column chromatography.

#### 4.2.2. Synthesis of Compound **4a**

Cinnamyl chloride (3 mmol) was dissolved in dry dichloromethane and mixed with methyl pyrrolidine-2 carboxylate hydrochloride (3.6 mmol) and DMAP (3.6 mmol). The mixture was stirred at ambient temperature overnight; it was washed successively with HCl (5%), NaHCO_3_ (5%), and a saturated NaCl solution; and the organic layer was dried over Na_2_SO_4_. Finally, the solvent was distilled off under a low pressure, followed by the purification of the final product via flash column chromatography.

#### 4.2.3. Synthesis of Compounds **1b**–**5b**

The corresponding amide of proline methyl ester (**1a**–**5a**) synthesized in the previous step was dissolved in 1,4-dioxane (20 mL) and an equal volume of an aqueous NaOH solution (5%) was added. The mixture was stirred for 45 min. Then, dioxane was removed under a reduced pressure, the aqueous phase was acidified using an HCl solution (10%), and it was extracted with ethyl acetate (3 × 20 mL). The combined organic fractions were dried over Na_2_SO_4_, and the solvent was evaporated under a reduced pressure.

#### 4.2.4. General Procedure for the Synthesis of Compounds **1c**–**5c**

The corresponding amide of proline (1 mmol) was dissolved in dry dichloromethane, using a small amount of DMF (up to 0.5 mL) to increase the solubility. Then, methyl 4-aminobutanoate hydrochloride (1.2 mmol), N,N-dimethylaminopyridine (DMAP, 1.2 mmol), and N,N dicyclohexylcarbodiimide (DCC, 1.2 mmol, after 15 min) were added, and the mixture was stirred overnight (at room temperature). The resulting mixture was purified with filtration and washed with HCl (5%), NaHCO_3_ (5%), and a saturated NaCl solution and dried over Na_2_SO_4_. Finally, the solvent was distilled off under a low pressure, followed by the purification of the final product via flash column chromatography.

(*E*)-methyl 1-(3-(4-hydroxy-3-methoxyphenyl)acryloyl)pyrrolidine-2-carboxylate (**1a**): Flash column chromatography (ethyl acetate/petroleum ether 3/1). Yellow powder, yield of 57%, mp. of 118–121 °C.

**IR (nujol)**: 3405 (O-H), 3217 (N-H), 1708 (C=O ester), 1630 (C=O amide), 1600, 1574 (C-C aromatic) cm^−1^.

**^1^H-NMR (CDCl_3_)**: 7.64 (d J: 15.4 Hz, 1H, Ph-C**H**=CH-), 7.10 (d J: 8.2 Hz, 1H, 6-aromatic) 6.98 (s, 1H, 2-aromatic), 6.90 (d J: 8.2 Hz, 1H, 5-aromatic), 6.58 (d J: 15.4 Hz, 1H, Ph-CH=C**H**-), 4.61 (dd J: 8.0, 3.2 Hz, 1H, 2-pyrrolidine), 3.91 (s, 3H, Ph-OCH_3_), 3.67–3.73, 3.82–3.89 (m, 2H, 5-pyrrolidine), 3.75 (s, 3H, -OCH_3_) 2.09–2.26 (m, 2H, 3-pyrrolidine), 1.53–1.79 (m, 2H, 4-pyrrolidine).

**^13^C-NMR (CDCl_3_)**: 172.25 (1C, -**C**OOCH_3_), 165.18 (1C, -**C**O-N), 147.47 (1C, 4-aromatic), 146.64 (1C, 3-aromatic), 143.09 (1C, Ph-**C**H=C), 127.99 (1C, 1-aromatic), 122.16 (1C, 6-aromatic), 115.30 (1C, Ph-CH=**C**H), 114.71 (1C, 5-aromatic), 109.95 (1C, 2 aromatic), 59.02 (1C, 2-pyrrolidine), 55.95 (1C, Ph-O**C**H_3_), 52.28 (1C, -O**C**H_3_), 46.94 (1C, 5-pyrrolidine), 29.19 (1C, 3-pyrrolidine), 24.86 (1C, 4-pyrrolidine) (See Supplementary File).

(*E*)-1-(3-(4-hydroxy-3-methoxyphenyl)acryloyl)pyrrolidine-2-carboxylic acid (**1b**): Yellow powder, yield of 93%, mp. of 169–172 °C).

**IR (nujol)**: 3405 (O-H), 3250 (COO-H), 3206 (N-H), 1732 (C=O carboxylic), 1633 (C=O amide), 1603, 1572 (C-C aromatic) cm^−1^.

**^1^H-NMR (CDCl_3_)**: 10.26 (s, 1H, -COOH), 7.76 (d J: 15.3 Hz, 1H, Ph-C**H**=CH-), 7.14 (d J: 8.2 Hz, 1H, 6-aromatic), 7.01 (s, 1H, 2-aromatic), 6.94 (d J: 8.2 Hz, 1H, 5-aromatic), 6.54 (d J: 15.3 Hz, 1H, Ph-CH=C**H**-), 4.74 (dd J: 7.9, 3.1 Hz, 1H, 2-pyrrolidine), 3.94 (s, 3H, Ph-OCH_3_), 3.69–3.80, (m, 2H, 5-pyrrolidine), 2.05–2.12 (m, 2H, 3-pyrrolidine), 1.83–2.01 (m, 2H, 4-pyrrolidine).

**^13^C-NMR (CDCl_3_)**: 171.79 (1C, -**C**OOH), 168.64 (1C, -**C**O-N), 148.23 (1C, 4-aromatic), 146.75 (1C, 3-aromatic), 145.58 (1C, Ph-**C**H=C), 126.91 (1C, 1-aromatic), 122.71 (1C, 6-aromatic), 114.90 (1C, Ph-CH=**C**H), 113.45 (1C, 5-aromatic), 110.31 (1C, 2 aromatic), 60.73 (1C, 2-pyrrolidine), 58.01 (1C, Ph-O**C**H_3_), 47.92 (1C, 5-pyrrolidine), 28.85 (1C, 3-pyrrolidine), 24.83 (1C, 4-pyrrolidine).

(*E*)-methyl 4-(1-(3-(4-hydroxy-3-methoxyphenyl)acryloyl)pyrrolidine-2-carboxamido)butanoate (**1c**): Flash column chromatography (ethyl acetate/acetone 3/1). Yellow powder, yield of 24%, mp. of 167–170 °C.

**IR (nujol)**: 3405 (O-H), 3215 (N-H), 1712 (C=O ester), 1634, 1627 (C=O amide), 1600, 1572 (C-C aromatic) cm^−1^.

**^1^H-NMR (CDCl_3_)**: 7.65(d J: 15.3 Hz, 1H, Ph-C**H**=CH-), 7.44 (s, 1H, -NH) 7.11 (d J: 8.2 Hz, 1H, 6-aromatic), 7.00 (s, 1H, 2-aromatic) 6.92 (d J: 8.2 Hz, 1H, 5-aromatic), 6.58 (d J: 15.3 Hz, 1H, Ph-CH=C**H**-), 4.72 (dd J: 7.9, 3.1 Hz, 1H, 2-pyrrolidine), 3.93 (s, 3H, Ph-OCH_3_), 3.60–3.63, 3.73–3.79 (m, 2H, 5-pyrrolidine), 3.64 (s, 3H, -OCH_3_), 3.21–3.31 (m, 2H, -NH-C**H_2_**-CH_2_-), 2.35 (t J: 7.4 Hz, 2H, -C**H_2_**-C=O) 2.09–2.18, 2.23–2.29 (m, 2H, 3-pyrrolidine), 1.77–1.88 (m, 4H, 4-pyrrolidine, -ΝH-CH_2_-C**H_2_**-).

**^13^C-NMR (CDCl_3_)**: 173.65 (1C, -**C**OOCH_3_), 171.29 (1C, -**C**O-NH), 166.64 (1C, -**C**O-N), 147.72 (1C, 4-aromatic), 146.27 (1C, 3-aromatic), 143.46 (1C, Ph-**C**H=C), 127.40 (1C, 1-aromatic), 122.33 (1C, 6-aromatic), 115.20 (1C, Ph-CH=**C**H), 114.80 (1C, 5-aromatic), 109.99 (1C, 2-aromatic), 60.00 (1C, 2-pyrrolidine), 55.97 (1C, Ph-O**C**H_3_), 51.80 (1C, -O**C**H_3_), 47.48 (1C, 5-pyrrolidine), 38.77 (1C, -NH-**C**H_2_), 31.38 (1C, -**C**H_2_-COOCH_3_), 26.97 (1C, 3-pyrroldine), 25.02 (1C, 4-pyrroldine), 24.72 (1C, -C**-C**H_2_-C).

Anal. calculated for C_20_H_26_N_2_O_6_: C, 61.53; H, 6.71; N, 7.18%. Found: C, 61.93; H, 7.08; N, 6.89%.

(*E*)-methyl 1-(3-(4-hydroxy-3,5-dimethoxyphenyl)acryloyl)pyrrolidine-2-carboxylate (**2a**): Flash column chromatography (ethyl acetate). Yellow oil, yield of 68%.

**IR (nujol):** 3415 (O-H), 3227 (N-H), 1711 (C=O ester), 1638 (C=O amide), 1602, 1575 (C-C aromatic) cm^−1^.

**^1^H-NMR (CDCl_3_)**: 7.62 (d J: 15.3 Hz, 1H, Ph-C**H**=CH-), 6.75 (s, 2H, aromatic), 6.57 (d J: 15.3 Hz, 1H, Ph-CH=C**H**-), 4.62 (dd J: 8.6, 4.1 Hz, 1H, 2-pyrrolidine), 3.91 (s, 6H, Ph-OCH_3_), 3.69–3.74, 3.85–3.89 (m, 2H, 5-pyrrolidine), 3.75 (s, 3H, -OCH_3_) 2.01–2.26 (m, 4H, 3,4-pyrrolidine).

**^13^C-NMR (CDCl_3_)**: 172.88 (1C, -**C**OOCH_3_), 165.01 (1C, -**C**O-N), 147.13 (2C, 3,5-aromatic), 143.27 (1C, Ph-**C**H=C), 136.70 (1C, 4-aromatic), 126.61 (1C, 1-aromatic), 115.75 (1C, Ph-CH=**C**H), 104.73 (2C, 2,6-aromatic), 59.01 (1C, 2-pyrrolidine), 56.36 (2C, Ph-O**C**H_3_), 52.23 (1C, -O**C**H_3_), 46.96 (1C, 5-pyrrolidine), 29.17 (1C, 3-pyrrolidine), 24.86 (1C, 4-pyrrolidine).

(*E*)-1-(3-(4-hydroxy-3,5-dimethoxyphenyl)acryloyl)pyrrolidine-2-carboxylic acid (**2b**): Yellow powder, yield of 86%, mp. of 94–98 °C.

**IR (nujol)**: 3405 (O-H), 3275 (COO-H), 3202 (N-H), 1736 (C=O carboxylic), 1632 (C=O amide), 1602, 1570 (C-C aromatic) cm^−1^.

**^1^H-NMR (CDCl_3_)**: 7.73 (d J: 15.3 Hz, 1H, Ph-C**H**=CH-), 6.76 (s, 2H, aromatic), 6.52 (d J: 15.3 Hz, 1H, Ph-CH=C**H**-), 4.72 (dd J: 8.6, 4.1 Hz, 1H, 2-pyrrolidine), 3.91 (s, 6H, Ph-OCH_3_), 3.76–3.87 (m, 2H, 5-pyrrolidine), 3.70 (s, 3H, -OH) 2.48–2.54 (m, 1H, 3-pyrrolidine), 1.96–2.12 (m, 3H, 3-pyrrolidine, 4-pyrrolidine).

**^13^C-NMR (CDCl_3_)**: 172.09 (1C, -**C**OOH), 168.03 (1C, -**C**O-N), 147.24 (2C, 3,5-aromatic), 145.54 (1C, Ph-**C**H=C), 137.23 (1C, 4-aromatic), 125.90 (1C, 1-aromatic), 113.95 (1C, Ph-CH=**C**H), 105.40 (2C, 2,6-aromatic), 60.50 (1C, 2-pyrrolidine), 56.40 (2C, Ph-O**C**H_3_), 48.83 (1C, 5-pyrrolidine), 27.20 (1C, 3-pyrrolidine), 24.79 (1C, 4-pyrrolidine).

(*E*)-methyl 4-(1-(3-(4-hydroxy-3,5-dimethoxyphenyl)-acryloyl)pyrrolidine-2-carboxamido)-butanoate (**2c**): Flash column chromatography (ethyl acetate/acetone 3/1). Yellow powder, yield of 58%, mp. of 158–159 °C.

**IR (nujol):** 3408 (O-H), 3223 (N-H), 1704 (C=O ester), 1644, 1631 (C=O amide), 1602, 1578 (C-C aromatic) cm^−1^.

**^1^H-NMR (CDCl_3_)**: 7.63 (d J:15.3 Hz, 1H, Ph-C**H**=CH-), 7.42 (s, 1H, -NH) 7.00 (s, 2H, aromatic), 6.57 (d J: 15.3 Hz, 1H, Ph-CH=C**H**-), 4.70 (dd J: 8.5, 4.1 Hz, 1H, 2-pyrrolidine), 3.92 (s, 6H, Ph-OCH_3_), 3.73–3.79 (m, 1H, 5-pyrrolidine), 3.64 (s, 3H, -OCH_3_), 3.19–3.36 (m, 3H, -NH-C**H_2_**-CH_2_-, 5-pyrrolidine), 2.34 (t J: 7.4 Hz, 2H, -C**H_2_**-C=O) 1.85–1.89, 2.01, 2.06 (m, 2H, 3-pyrrolidine), 1.77–1.85 (m, 4H, 4-pyrroldine, -ΝH-CH_2_-C**H_2_**-).

**^13^C-NMR (CDCl_3_)**: 173.64 (1C, -**C**OOCH_3_), 171.24 (1C, -**C**O-NH), 166.50 (1C, -**C**O-N), 147.20 (2C, 3,5-aromatic), 143.68 (1C, Ph-**C**H=C), 136.96 (1C, 4-aromatic), 126.35 (1C, 1-aromatic), 115.50 (1C, Ph-CH=**C**H), 105.09 (2C, 2,6-aromatic), 60.01 (1C, 2-pyrrolidine), 56.41 (2C, Ph-O**C**H_3_), 56.35 (1C, -O**C**H_3_), 47.55 (1C, 5-pyrrolidine), 38.80 (1C, -NH-**C**H_2_), 31.41 (1C, -**C**H_2_-COOCH_3_), 26.94 (1C, 3-pyrrolidine), 25.01 (1C, 4-pyrrolidine), 24.72 (1C, -C**-C**H_2_-C).

Anal. calculated for C_21_H_28_N_2_O_7_: C, 59.99; H, 6.71; N, 6.66%. Found: C, 60.08; H, 6.88; N, 6.54%.

(*E*)-methyl 1-(3-(3,4-dimethoxyphenyl)acryloyl)pyrrolidine-2-carboxylate (**3a**): Flash column chromatography (ethyl acetate/petroleum ether 3/1). Colorless oil, yield of 78%.

**IR (nujol)**: 3226 (N-H), 1709 (C=O ester), 1636 (C=O amide), 1601, 1568 (C-C aromatic) cm^−1^.

**^1^H-NMR (CDCl_3_)**: 7.65 (d J: 15.5 Hz, 1H, Ph-C**H**=CH-), 7.10 (d J: 8.2 Hz, 1H, 6-aromatic) 7.02 (s, 1H, 2-aromatic), 6.84 (d J: 8.2 Hz, 1H, 5-aromatic), 6.59 (d J: 15.4 Hz, 1H, Ph-CH=C**H**-), 4.61 (dd J: 8.5, 4.2 Hz, 1H, 2-pyrrolidine), 3.90 (s, 3H, Ph-OCH_3_), 3.89 (s, 3H, Ph-OCH_3_) 3.68–3.72, 3.83–3.88 (m, 2H, 5-pyrrolidine), 3.74 (s, 3H, -OCH_3_) 2.09–2.27 (m, 2H, 3-pyrrolidine), 1.93–2.06 (m, 2H, 4-pyrrolidine).

**^13^C-NMR (CDCl_3_)**: 172.88 (1C -**C**OOCH_3_), 165.11 (1C -**C**O-N), 150.67 (1C, 4-aromatic), 149.07 (1C, 3-aromatic), 142.91 (1C, Ph-**C**H=C), 128.07 (1C, 1-aromatic), 122.09 (1C, 6-aromatic), 115.67 (1C, Ph-CH=**C**H), 111.06 (1C, 2-aromatic), 110.01 (1C, 5-aromatic), 59.02 (1C, 2-pyrrolidine), 55.93 (2C, Ph-OCH_3_), 52.21 (1C, -OCH_3_), 46.94 (1C, 5-pyrrolidine), 29.16 (1C, 3-pyrrolidine), 24.84 (1C, 4-pyrrolidine).

(*E*)-1-(3-(3,4-dimethoxyphenyl)acryloyl)pyrrolidine-2-carboxylic acid (**3b**): White powder, yield of 95%, mp. of 186–188 °C.

**IR (nujol)**: 3285 (COO-H), 3217 (N-H), 1732 (C=O carboxylic), 1631 (C=O amide), 1599, 1567 (C-C aromatic) cm^−1^.

**^1^H-NMR (CDCl_3_)**: 7.78 (d J: 15.3 Hz, 1H, Ph-C**H**=CH-), 7.16 (d J: 8.2 Hz, 1H, 6-aromatic) 7.04 (s, 1H, 2-aromatic), 6.88 (d J: 8.2 Hz, 1H, 5-aromatic), 6.55 (d J: 15.3 Hz, 1H, Ph-CH=C**H**-), 4.74 (dd J: 8.5, 4.2 Hz, 1H, 2-pyrrolidine), 3.92 (s, 6H, Ph-OCH_3_), 3.65–3.71, 3.75–3.82 (m, 2H, 5-pyrrolidine), 2.06–2.15 (m, 2H, 3-pyrrolidine), 1.92–2.01 (m, 2H, 4-pyrrolidine).

**^13^C-NMR (CDCl_3_)**: 171.49 (1C, -**C**OOH), 168.57 (1C, -**C**O-N), 148.23 (1C, 4-aromatic), 146.75 (1C, 3-aromatic), 145.58 (1C, Ph-**C**H=C), 126.91 (1C, 1-aromatic), 122.71 (1C, 6-aromatic), 114.90 (1C, Ph-CH=**C**H), 113.45 (1C, 2-aromatic), 110.31 (1C, 5-aromatic), 60.73 (1C, 2-pyrrolidine), 58.11 (2C, Ph-O**C**H_3_), 47.92 (1C, 5-pyrrolidine), 28.85 (1C, 3-pyrrolidine), 24.83 (1C, 4-pyrrolidine).

(*E*)-methyl 4-(1-(3-(3,4-dimethoxyphenyl)acryloyl)pyrrolidine-2-carboxamido)butanoate (**3c**): Flash column chromatography (ethyl acetate/petroleum ether 3/1). Yellow powder, yield of 78% mp. of 158–159 °C.

**IR (nujol)**: 3220 (N-H), 1715 (C=O ester), 1631, 1619 (C=O amide), 1598, 1564 (C-C aromatic) cm^−1^.

**^1^H-NMR (CDCl_3_)**: 7.68 (d J: 15.4 Hz, 1H, Ph-C**H**=CH-), 7.45 (s, 1H, -NH) 7.14 (d J: 8.2 Hz, 1H, 6-aromatic), 7.05 (s, 1H, 2-aromatic) 6.88 (d J: 8.2 Hz, 1H, 5-aromatic), 6.61 (d J: 15.4 Hz, 1H, Ph-CH=C**H**-), 4.72 (d J: 7.9 Hz, 1H, 2-pyrrolidine), 3.93 (s, 6H, Ph-OCH_3_), 3.92 (s, 6H, Ph-OCH_3_), 3.73–3.79 (m, 1H, 5-pyrrolidine), 3.65 (s, 3H, -OCH_3_), 3.20–3.35 (m, 3H, -NH-C**H_2_**-CH_2_-, 5-pyrrolidine), 2.35 (t J: 7.4 Hz, 2H, -C**H_2_**-C=O) 2.01, 2.20 (m, 2H, 3-pyrrolidine), 1.75–1.88 (m, 4H, 4-pyrrolidine, -ΝH-CH_2_-C**H_2_**-).

**^13^C-NMR (CDCl_3_)**: 173.66 (1C, -**C**OOCH_3_), 171.27 (1C, -**C**O-NH), 166.61 (1C, -**C**O-N), 150.91 (1C, 4-aromatic), 149.16 (1C, 3-aromatic), 143.31 (1C, Ph-**C**H=C), 127.85 (1C, 1-aromatic), 122.22 (1C, 6-aromatic), 115.52 (1C, Ph-CH=**C**H), 111.12 (1C, 5-aromatic), 110.06 (1C, 2-aromatic), 60.01 (1C, 2-pyrrolidine, 55.96 (2C, Ph-O**C**H_3_), 51.61 (1C, -O**C**H_3_), 47.51 (1C, 5-pyrrolidine), 38.77 (1C, -NH-**C**H_2_), 31.40 (1C, -**C**H_2_-COOCH_3_), 26.98 (1C, 3-pyrrolidine), 25.03 (1C, 4-pyrrolidine), 24.73 (1C, -C**-C**H_2_-C).

Anal. calculated for C_21_H_28_N_2_O_6_: C, 62.36; H, 6.98; N, 6.93%. Found: C, 62.48; H, 7.36; N, 6.99%.

Methyl 1-cinnamoylpyrrolidine-2-carboxylate (**4a**): Flash column chromatography (ethyl acetate/petroleum ether 1/1). White powder, yield of 90%, mp. of 88–89 °C.

**IR (nujol)**: 3222 (N-H), 1709 (C=O ester), 1628 (C=O amide), 1601, 1572 (C-C aromatic) cm^−1^.

**^1^H-NMR (CDCl_3_)**: 7.72 (d J: 15.4 Hz, 1H, Ph-C**H**=CH-), 7.49–7.53 (m, 2H, aromatic), 7.32–7.43 (m, 3H, aromatic), 6.75 (d J: 15.4 Hz, 1H, Ph-CH=C**H**-), 4.62 (dd J: 7.7, 3.1 Hz, 1H, 2-pyrrolidine), 3.75 (s, 3H, -OCH_3_) 3.69–3.73, 3.84–3.88 (m, 2H, 5-pyrrolidine), 2.09–2.17, 2.20–2.27 (m, 2H, 3-pyrrolidine), 1.98–2.08 (m, 2H, 4-pyrrolidine).

**^13^C-NMR (CDCl_3_)**: 172.81 (1C, -**C**OOCH_3_), 164.86 (1C, -**C**O-N), 142.92 (1C, Ph-**C**H=C), 135.10 (1C, 1-aromatic), 129.74 (1C, 4-aromatic), 128.77 (2C, 3,5-aromatic), 127.92 (2C, 2,6-aromatic), 117.92 (1C, Ph-CH=**C**H), 59.04 (1C, 2-pyrrolidine), 52.23 (1C, -O**C**H_3_), 46.95 (1C, 5-pyrrolidine), 29.16 (1C, 3-pyrrolidine), 24.95 (1C, 4-pyrrolidine).

1-cinnamoylpyrrolidine-2-carboxylic acid (**4b**): White powder, yield of 92%, mp. of 180–182 °C.

**IR (nujol)**: 3287 (COO-H), 3214 (N-H), 1732 (C=O carboxylic), 1634 (C=O amide), 1602, 1571 (C-C aromatic) cm^−1^.

**^1^H-NMR (CDCl_3_)**: 7.62 (d J: 15.5 Hz, 1H, Ph-C**H**=CH-), 7.44–7.53 (m, 2H, aromatic), 7.29–7.43 (m, 3H, aromatic), 6.75 (d J: 15.5 Hz, 1H, Ph-CH=C**H**-), 4.54 (dd J: 8.1, 3.3 Hz, 1H, 2-pyrrolidine), 3.57–3.73, 3.76–3.88 (m, 2H, 5-pyrrolidine), 1.99–2.24 (m, 4H, 3,4-pyrrolidine).

**^13^C-NMR (CDCl_3_)**: 178.48 (1C, -**C**OOH), 169.69 (1C, -**C**O-N), 147.21 (1C, Ph-**C**H=C), 139.72 (1C, 1-aromatic), 134.51 (1C, 4-aromatic), 133.55 (2C, 3,5-aromatic), 132.61 (2C, 2,6-aromatic), 123.02 (1C, Ph-CH=**C**H), 63.98 (1C, 2-pyrrolidine), 51.84 (1C, 5-pyrrolidine), 33.67 (1C, 3-pyrrolidine), 29.46 (1C, 2-pyrrolidine).

(E)-methyl 4-(1-cinnamoylpyrrolidine-2-carboxamido)butanoate (**4c**): Flash column chromatography (ethyl acetate). White powder, yield of 75%, mp. of 76–78 °C.

**IR (nujol)**: 3215 (N-H), 1716 (C=O ester), 1634, 1623 (C=O amide), 1605, 1577 (C-C aromatic) cm^−1^.

**^1^H-NMR (CDCl_3_)**: 7.72 (d J: 15.5 Hz, 1H, Ph-C**H**=CH-), 7.50–7.58 (m, 2H, aromatic), 7.33–7.45 (m, 3H, aromatic), 6.75 (d J: 15.5 Hz, 1H, Ph-CH=C**H**-), 4.70 (dd J: 8.0, 3.3 Hz, 1H, 2-pyrrolidine), 3.64 (s, 3H, -OCH_3_), 3.58–3.62, 3.73–3.79 (m, 2H, 5-pyrrolidine), 3.17–3.36 (m, 2H, -NH-C**H_2_**-CH_2_-), 2.34 (t J: 7.4 Hz, 2H, -C**H_2_**-C=O), 2.00–2.20 (m, 2H, 3-pyrrolidine), 1.77–1.90 (m, 4H, 4-pyrrolidine, -NH-CH_2_-C**H_2_**-).

**^13^C-NMR (CDCl_3_)**: 173.64 (1C, -**C**OOCH_3_), 171.18 (1C, -**C**O-NH), 166.31 (1C, -**C**O-N), 143.28 (1C, Ph-**C**H=C), 134.86 (1C, 1-aromatic), 129.98 (1C, 4-aromatic), 128.85 (2C, 3,5-aromatic), 127.97 (2C, 2,6-aromatic), 117.83 (1C, Ph-CH=**C**H), 60.05 (1C, 2-pyrrolidine), 51.59 (1C, -O**C**H_3_), 47.51 (1C, 5-pyrrolidine), 38.79 (1C, -NH-**C**H_2_), 31.39 (1C, -**C**H_2_-COOCH_3_), 27.05 (1C, 3-pyrrolidine), 25.02 (1C, 4-pyrrolidine), 24.70 (1C, -C**-C**H_2_-C).

Anal. calculated for C_19_H_24_N_2_O_4_: C, 62.53; H, 6.89; N, 4.56%. Found: C, 62.79; H, 7.12; N, 4.66%.

Methyl 1-(6-hydroxy-2,5,7,8-tetramethylchroman-2-carbonyl)pyrrolidine-2-carboxylate (**5a**): Flash column chromatography (ethyl acetate/petroleum ether 2/3). Colorless oil, yield of 73%.

**IR (nujol)**: 3401 (O-H), 3229 (N-H), 1720 (C=O ester), 1633 (C=O amide), 1608, 1570 (C-C aromatic) cm^−1^.

**^1^H-NMR (CDCl_3_)**: 4.36 (dd J: 11.1, 5.6 Hz, 1H, 2-pyrrolidine), 3.41–3.48, 4.04–4.16 (m, 2H, 5-pyrrolidine), 3.58, 3.71 (s, 3H, -OCH_3_), 2.49–2.73 (m, 4H, 3,4-chroman), 2.17 (s, 3H, Ph-CH_3_), 2.16 (s, 3H, Ph-CH_3_), 2.06, 2.10 (s, 3H, Ph-CH_3_), 1.68–1.96 (m, 4H, 3,4-pyrrolidine), 1.54, 1.61 (s, 3H, 2-CH_3_).

**^13^C-NMR (CDCl_3_)**: 173.19/172.95 (1C, -**C**OOCH_3_), 172.79/172.50 (1C, -**C**O-N), 145.31/145.23 (1C, 9-chroman), 144.68/144.44 (1C, 6-chroman), 121.54/121.50 (1C, 5-chroman), 121.29/121.20 (1C, 8-chroman), 118.94/118.77 (1C, 7-chroman), 118.24/118.20 (1C, 10-chroman), 78.81/78.67 (1C, 2-chroman), 60.80/60.39 (1C, 2-pyrrolidine), 52.01/51.84 (1C, -O**C**H_3_), 48.12/47.98 (1C- 5-pyrrolidine), 30.52/30.51 (1C, 3-pyrrolidine), 27.81/27.58 (1C, 3-chroman), 26.06/25.43 (1C, 4-pyrrolidine), 24.96/24.41 (1C, 4-chroman), 21.06/20.67 (1C, 2-**C**H_3_), 12.24/12.20 (1C, 5-**C**H_3_), 12.16/12.13 (1C, 8-**C**H_3_), 11.28/11.25 (1C, 7-**C**H_3_).

2-(6-hydroxy-2,5,7,8-tetramethylchroman-2-carbonyl)pyrrolidine-1-carboxylic acid (**5b**): White powder, yield of 97%, mp. of 135–138 °C.

**IR (nujol)**: 3401 (O-H), 3280 (COO-H), 3220 (N-H), 1741 (C=O carboxylic), 1639 (C=O amide), 1606, 1570 (C-C aromatic) cm^−1^.

**^1^H-NMR (CDCl_3_)**: 4.42 (dd J: 11.1, 5.6 Hz, 1H, 2-pyrrolidine), 3.38–3.50, 4.03–4.09 (m, 2H, 5-pyrrolidine), 2.51–2.73 (m, 4H, 3,4-chroman), 2.16 (s, 3H, Ph-CH_3_), 2.14 (s, 3H, Ph-CH_3_), 2.04, 2.08 (s, 3H, Ph-CH_3_), 1.63–2.01 (m, 4H, 3,4-pyrrolidine), 1.56, 1.61 (s, 3H, 2-CH_3_).

**^13^C-NMR (CDCl_3_)**: 175.58/175.09 (1C, -**C**OOH), 174.80/174.27 (1C, -**C**O-N), 145.42/145.36 (1C, 9-chroman), 144.51/144.28 (1C, 6-chroman), 121.51 (1C, 5-chroman), 121.40 (1C, 8-chroman), 119.10/119.02 (1C, 7-chroman), 118.08/118.03 (1C, 10-chroman), 78.89/78.78 (1C, 2-chroman), 60.86/60.42 (1C, 2-pyrrolidine), 48.44/48.31 (1C, 5-pyrrolidine), 30.61/30.46 (1C, 3-pyrrolidine), 27.04/26.97 (1C, 3-chroman), 25.94/25.50 (1C, 4-pyrrolidine), 25.01/24.38 (1C, 4-chroman), 21.02/20.67 (1C, 2-**C**H_3_), 12.25/12.21 (1C, 5-**C**H_3_), 12.15/12.11 (1C, 8-**C**H_3_), 11.27/11.18 (1C, 7-**C**H_3_).

Methyl 4-(1-(6-hydroxy-2,5,7,8-tetramethylchroman-2-carbonyl)pyrrolidine-2-carboxamido)-butanoate (**5c**): Flash column chromatography (ethyl acetate/acetone 8/1). Yellow powder, yield of 45%, mp. of 59–61 °C. 

**IR (nujol)**: 3401 (O-H), 3227 (N-H), 1712 (C=O ester), 1638, 1628 (C=O amide), 1608, 1570 (C-C aromatic) cm^−1^.

**^1^H-NMR (CDCl_3_)**: 4.42 (dd J: 11.1, 5.6 Hz, 1H, 2-pyrrolidine), 3.66, 3.68 (s, 3H, -OCH_3_), 3.24–3.29, 3.43–3.52 (m, 2H, 5-pyrrolidine), 2.70–2.79, 3.08–3.16 (m, 2H, -NH-C**H_2_**-CH_2_-) 2.49–2.68 (m, 4H, 3,4-chroman), 2.34 (t J: 7.4 Hz, 2H, -C**H_2_**-C=O), 2.18, 2.20 (s, 3H, Ph-CH_3_), 2.14, 2.15 (s, 3H, Ph-CH_3_), 2.04, 2.08 (s, 3H, Ph-CH_3_), 1.89–2.02 (m, 2H, 3-pyrrolidine), 1.66–1.84 (m, 2H, 4-pyrrolidine), 1.58, 1.61 (s, 3H, 2-CH_3_).

**^13^C-NMR (CDCl_3_)**: 174.40 (1C, -**C**OOCH_3_), 173.64 (1C, -**C**O-NH), 171.42 (1C, -**C**O-N), 145.83 (1C, 9-chroman), 144.81 (1C, 6-chroman), 123.45 (1C, 5-chroman), 121.75/121.40 (1C, 8-chroman), 120.68/120.32 (1C, 7-chroman), 118.16 (1C, 10-chroman), 78.75 (1C, 2-chroman), 61.47/60.96 (1C, 2-pyrrolidine), 51.90/51.61 (1C, -O**C**H_3_), 48.26/48.17 (1C, 5-pyrrolidine), 38.68/38.50 (1C, -NH-**C**H_2_), 31.78/31.30 (1C, -**C**H_2_-COOCH_3_), 31.09/30.85 (3-pyrrolidine), 27.99 (3-pyrrolidine), 25.76/25.52 (4-pyrrolidine), 24.75/24.60 (4-chroman), 24.38/24.29 (1C, -C**-C**H_2_-C), 21.48/20.97 (1C, 2-**C**H_3_), 12.39 (1C, 5-**C**H_3_), 12.25/12.13 (1C, 8-**C**H_3_), 11.54/11.28 (1C, 7-**C**H_3_).

Anal. calculated for C_24_H_34_N_2_O_6_: C, 64.55; H, 7.67; N, 6.66%. Found: C, 64.38; H, 7.29; N, 6.13%.

### 4.3. Biological Evaluation

κ-Carrageenan and soybean lipoxygenase type I-B were purchased from Sigma (St. Louis, MO, USA). For the in vivo experiments, Wistar rats (180–220 g, 3–4 months old) were kept at the Centre of the School of Veterinary Medicine (EL54 BIO42), Aristotle University of Thessaloniki, which is registered by the official state veterinary authorities (presidential degree 56/2013, in harmonization with European Directive 2010/63/EEC). The experimental protocols were approved by the Animal Ethics Committee of the Prefecture of Central Macedonia (No. 270079/2500).

#### 4.3.1. In Vitro Lipid Peroxidation Inhibition

The rat liver microsomal fraction was inactivated with heat at 90 °C for 90 s, and ascorbic acid (0.2 mM) and Fe_2_SO_4_ (10 μΜ) were added for the induction of its peroxidation. The compounds dissolved in dimethylsulfoxide (DMSO) were added (concentrations of 1 μΜ–1 mM). Aliquots were removed from the reaction mixture for 45 min (37 °C). The induction of peroxidation was assessed at 535/600 nm as 2-thiobarbituric acid-reactive material. None of the solvents or compounds affected the assay [28].

#### 4.3.2. In Vitro Interaction with the Stable Free Radical 1,1-Diphenyl-2-Picrylhydrazyl (DPPH)

The tested compounds, dissolved in absolute ethanol at final concentrations of 25 up to 200 μΜ, were mixed with DPPH and dissolved in the same solvent at a final concentration of 200 μΜ. The absorbance at 517 nm was recorded every 5 min for 30 min [41].

#### 4.3.3. In Vitro Protein Glycation Inhibition

Bovine serum albumin (BSA, 4 mg/mL) was incubated with fructose (250 mM), Cu^2+^ (10 μΜ), and NaN_3_ (0.015%) in a 120 mM phosphate buffer (pH of 7.4) at 37 °C for 72 h. The purification of the glycation product was based on protein precipitation and washing with trichloroacetic acid (TCA). The concentration of the glycation-modified protein was estimated by fluorescence measurements (excitation of 340 nm, emission of 410 nm). Incubations performed under the same conditions, yet in the absence of fructose, were used as a control. All the fluorescence measurements were expressed relatively to the standard quinine sulphate solution (1 μg/mL) [28]. All compounds were stable during the experiment.

#### 4.3.4. In Vitro Evaluation of Acetylcholinesterase Activity

The tested compounds, dissolved in 60% ethanol, were mixed with brain homogenate (20 mg of tissue per mL) from untreated rats (in 0.1 M phosphate buffer with a pH of 8) and acetylthiocholine (0.5 mM). The enzyme activity was assessed by evaluating the reaction product of the liberated thiocholine with 5,5-dithio-bis-(2-nitrobenzoic acid (DTNB) at 412 nm. The solvent system was examined and did not interfere with the assay [42].

#### 4.3.5. In Vitro Evaluation of Lipoxygenase Activity

The reaction mixture (total volume of 3 mL) contained 100 μL of the test compounds (100 μM) or the solvent (absolute ethanol, control), 200 μL of soybean LOX (250 u/mL, in a 0.9% NaCl solution), and 2.6 mL of Tris–HCl buffer with a pH of 9.0. The reaction was initiated by the addition of 100 μL sodium linoleate (100 μM) in the sample mixture (with an equal volume of buffer added to the reference solution). The reaction was followed for 7 min at 28 °C, and the absorbance (234 nm) of a conjugated diene structure was recorded. The performance of the assay was verified using NDGA as a reference [43].

#### 4.3.6. In Vivo Evaluation of Anti-Inflammatory Activity

The examined compounds were suspended in water with a small amount of Tween 80 and administered i.p. (0.15 mmol/kg) to the rats, just after an i.d. injection of 0.1 mL of an aqueous carrageenan solution (1% *w*/*v*) in the rear paw of the rats. The produced edema was estimated after 3.5 h as the paw weight increased [44].

#### 4.3.7. Statistical Analysis

All the tests in the in vitro assays were performed in triplicate and the standard deviations were always no more than 10% of the mean values (as calculated using MS Excel 2016). In the in vivo assay, the statistical significance of the results was estimated using *p* values, which were calculated according to Student’s *t*-test (https://www.graphpad.com/quickcalcs/ttest1.cfm) (accessed on 21 March 2024).

## 5. Conclusions

It is evident from the literature that oxidative stress and inflammation play a significant role in the development and progression of neurodegenerative conditions [2,3,4,5,6,7,8,9,10,11,12]. The involvement of various biochemical pathways in the pathogenesis of AD makes its radical treatment difficult, and the current treatment options are useful for alleviating only the symptoms. Multi-acting compounds could be of use in the therapeutic regimens of AD, since they affect many pathways associated with its pathophysiology.

In our work, we suggested the design and synthesis of a series of proline and GABA analogues containing antioxidant and/or anti-inflammatory moieties, such as ferulic acid, cinnamic acid, and Trolox. The Trolox derivative **5c** was a very strong lipid peroxidation inhibitor (IC_50_ of 8 μΜ) and an effective DPPH radical scavenger. Compounds **1c** and **2c** were weaker antioxidants, as their lipid peroxidation inhibition and DPPH-reducing capacity showed, although they demonstrated a higher inhibitory effect against protein glycation than compound **5c**. In addition, compounds **1c**–**3c** were moderate AchE inhibitors, while **4c**, with no aromatic substituents, and **5c**, with the bulky chroman group, could not inhibit the enzyme. Finally, all the compounds could inhibit LOX up to 40% at 100 μΜ and reduce rat paw edema. Compound **5c**, with great antioxidant properties, and **3c**, with no antioxidant capacity, were the most active against paw edema, indicating that inflammation is a complex procedure and various signaling pathways are involved in it. In conclusion, our results indicate that compounds with combined molecular characteristics could yield pluripotent derivatives that could act as such or as lead compounds towards the evolution of effective treatments for degenerative conditions such as neurodegenerative disorders.

## Figures and Tables

**Table 1:** Effect of compounds **1c**–**5c** and Trolox on lipid peroxidation.

Compound	IC_50_ (μΜ)
**1c**	839
**2c**	290
**3c**	-
**4c**	-
**5c**	8
**Trolox**	25

After 45 min of incubation. Trolox: 6-hydroxy-2,5,7,8-tetramethylchroman-2-carboxylic acid. All determinations were performed in triplicate and the standard deviation was always within ±10% of the mean value; -: inactive.

**Table 2:** Interaction of compounds **1c**–**5c** and Trolox, at various concentrations, with DPPH (200 μΜ).

Compound	% Interaction with DPPH			
	200 μΜ	100 μΜ	50 μΜ	25 μΜ
**1c**	58	37	24	10
**2c**	85	64	36	17
**3c**	-	-	-	-
**4c**	-	-	-	-
**5c**	89	87	54	28
**Trolox**	92	90	38	19

After 30 min of incubation. Trolox: 6-hydroxy-2,5,7,8-tetramethylchroman-2-carboxylic acid. All determinations were performed in triplicate and the standard deviation was always within ±10% of the mean value; -: inactive.

**Table 3:** Inhibitory activity of compounds **1c**–**5c** and aminoguanidine (concentration of 1mM) against protein glycation.

Compound	% Inhibition
**1c**	41
**2c**	48
**3c**	-
**4c**	-
**5c**	37
**Aminoguanidine**	56

After 72 h of incubation. All determinations were performed three times and the standard deviation was always no more than 10% of the mean value; -: inactive.

**Table 4:** Inhibitory activity of compounds **1c**–**5c** and physostigmine against acetylcholinesterase and their molecular volume, as calculated by Molinspiration.

Compound	IC_50_ (μΜ)	Molecular Volume (Å^3^) ^a^
**1c**	219	359.50
**2c**	298	385.05
**3c**	284	377.03
**4c**	-	325.94
**5c**	-	421.04
**Physostigmine**	0.15	

After 7 min of incubation. All determinations were performed three times and the standard deviation was always no more than 10% of the mean value; -: inactive; ^a^: calculated by Molinspiration.

**Table 5:** Inhibitory activity of compounds **1c**–**5c** (concentration of 100 μM) and NDGA against soybean lipoxygenase and their clogP values.

Compound	% Inhibition	clogP
**1c**	36	1.63
**2c**	40	1.41
**3c**	32	2.10
**4c**	40	2.44
**5c**	26	3.89
**NDGA**	94	

After 7 min of incubation. All determinations were performed three times and the standard deviation was always no more than 10% of the mean value; NDGA: nordihydroguaiaretic acid; clogP: calculated by ChemDraw 12.0.

**Table 6:** Effect of compounds **1c**–**5c**, naproxen, and ibuprofen on carrageenan-induced rat paw edema.

Compound	% Edema Reduction
**1c**	40 **
**2c**	49 ***
**3c**	55 **
**4c**	28 ***
**5c**	53 ***
**Naproxen**	11 *
**Ibuprofen**	36 *

The effect is expressed as the % edema reduction in comparison to controls, who received carrageenan only. All compounds were administered i.p. at 0.15 mmol/kg of body weight. Significant difference from control: * *p* < 0.01, ** *p* < 0.005, *** *p* < 0.001 (Student’s *t*-test).

## Data Availability

The data are available from the corresponding author upon a relevant request.

## Supplementary Materials

The following supporting information can be downloaded at: https://www.mdpi.com/article/10.3390/molecules29163763/s1, NMR spectra.
