# Supplementary material for: Trolox, Ferulic, Sinapic, and Cinnamic Acid Derivatives of Proline and GABA with Antioxidant and/or Anti-Inflammatory Properties
Source: Molecules. 2024 Aug 8;29(16):3763. doi: 10.3390/molecules29163763 (PMC11356873; doi:10.3390/molecules29163763)

(*E*)-methyl 1-(3-(4-hydroxy-3-methoxyphenyl)acryloyl)pyrrolidine-2-carboxylate (**1a**)

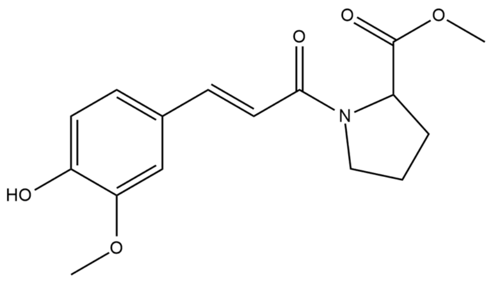

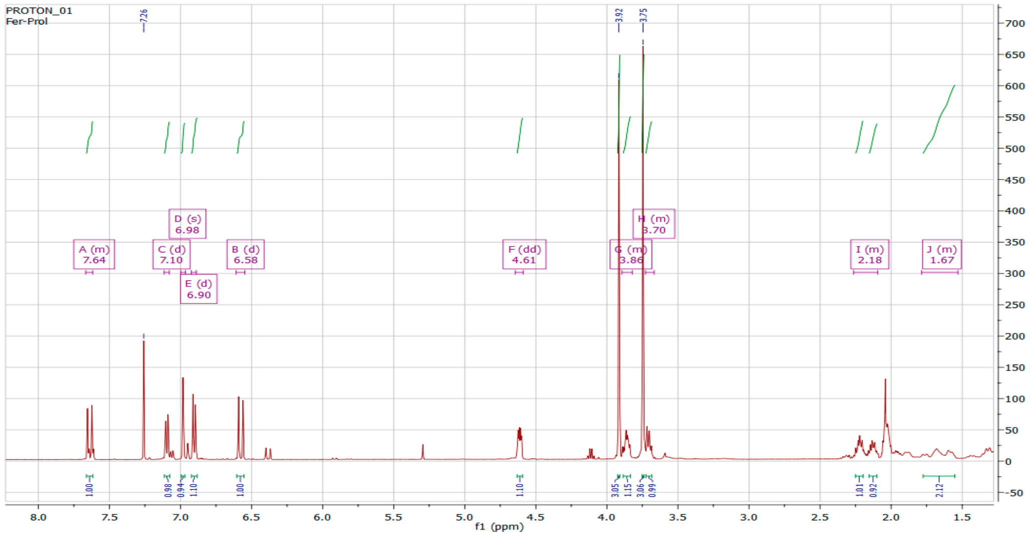

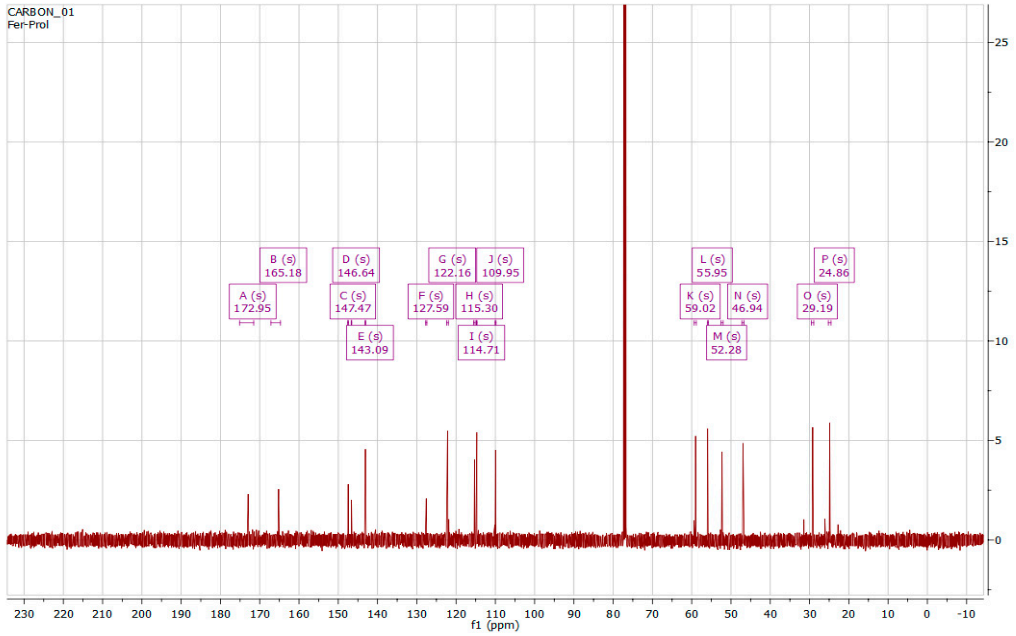

(*E*)-1-(3-(4-hydroxy-3-methoxyphenyl)acryloyl)pyrrolidine-2-carboxylic acid (**1b**)

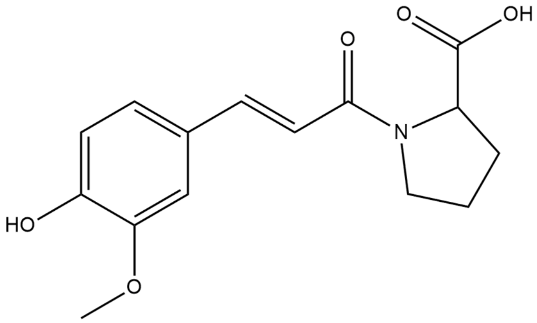

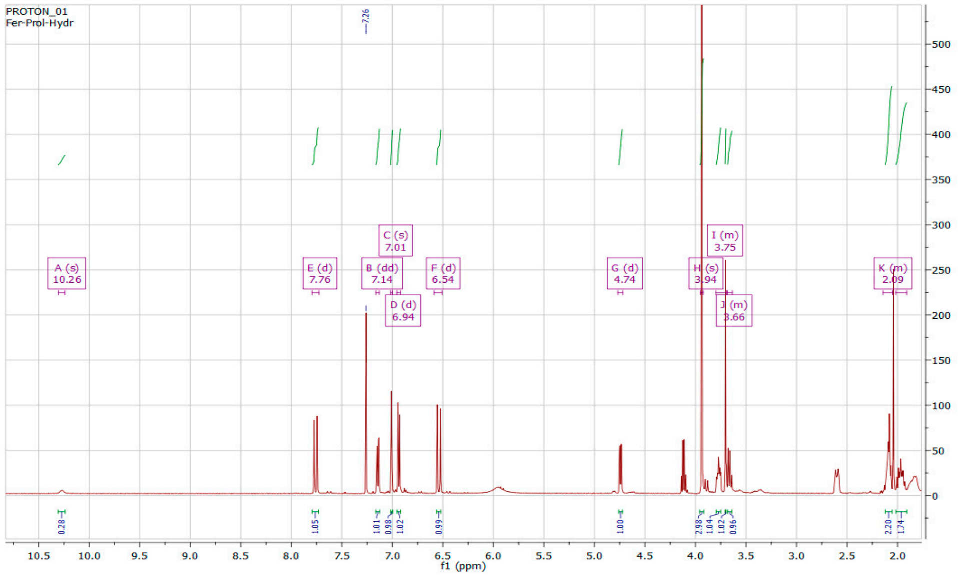

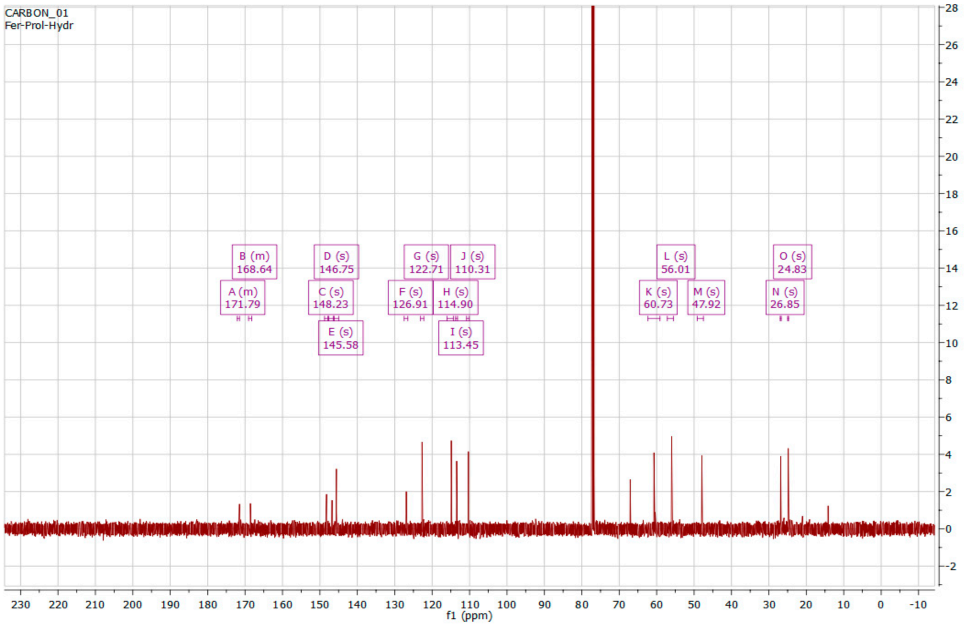

(*E*)-methyl 4-(1-(3-(4-hydroxy-3-methoxyphenyl)acryloyl)pyrrolidine-2-carboxamido)butanoate (**1c**)

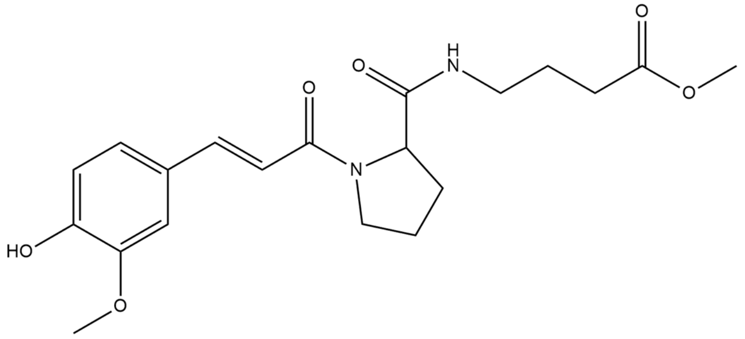

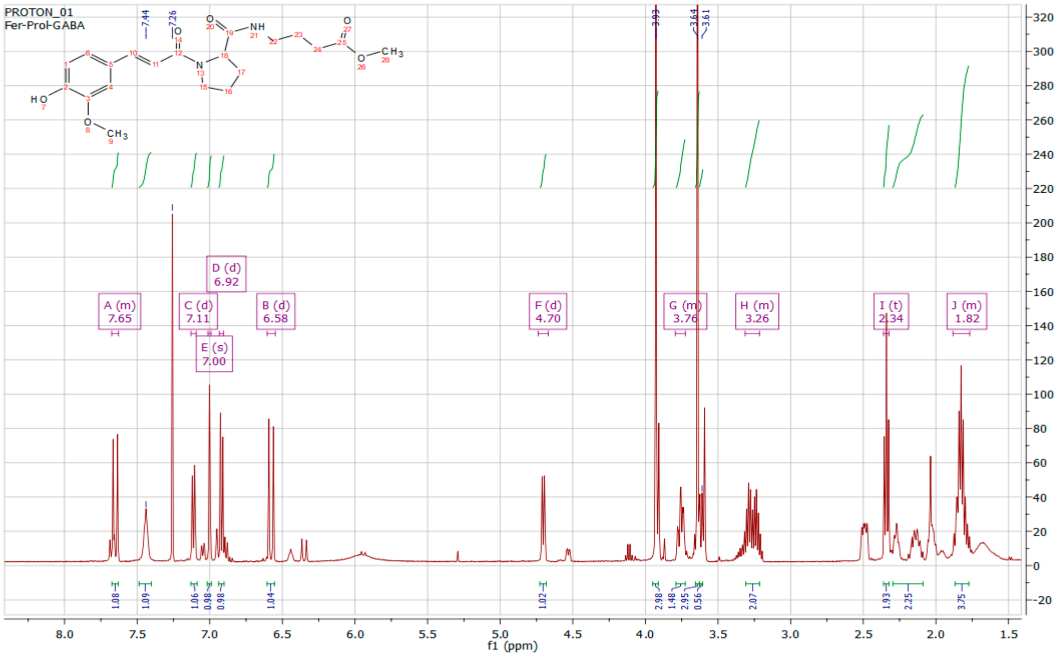

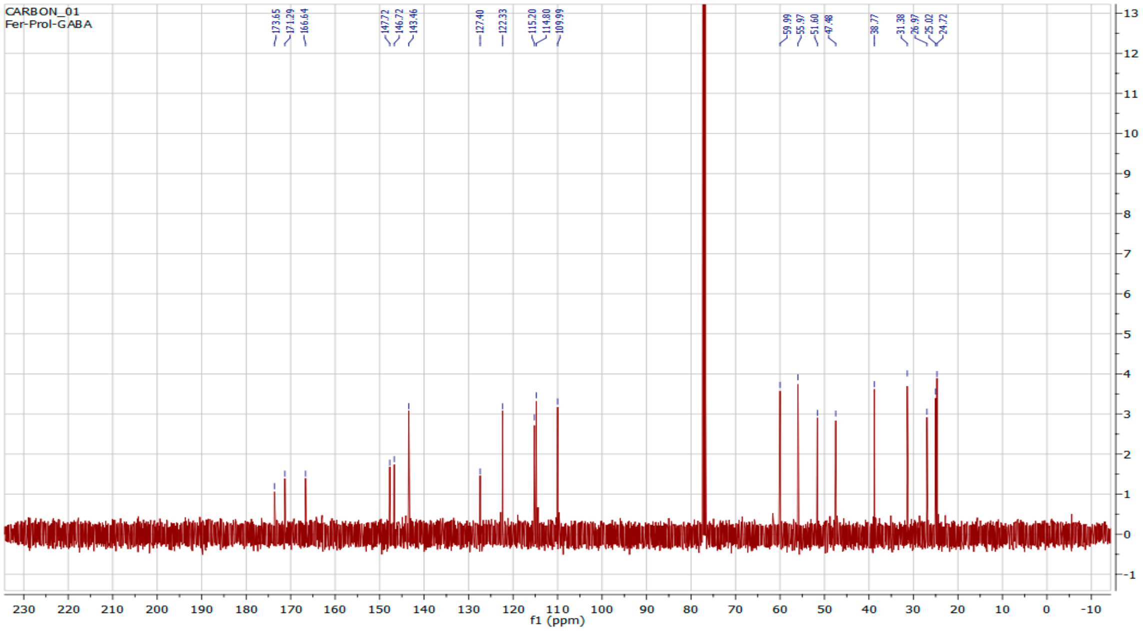

(E)-methyl 1-(3-(4-hydroxy-3,5-dimethoxyphenyl)acryloyl)pyrrolidine-2-carboxylate (**2a**)

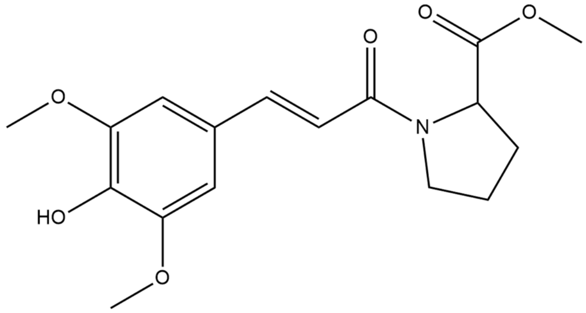

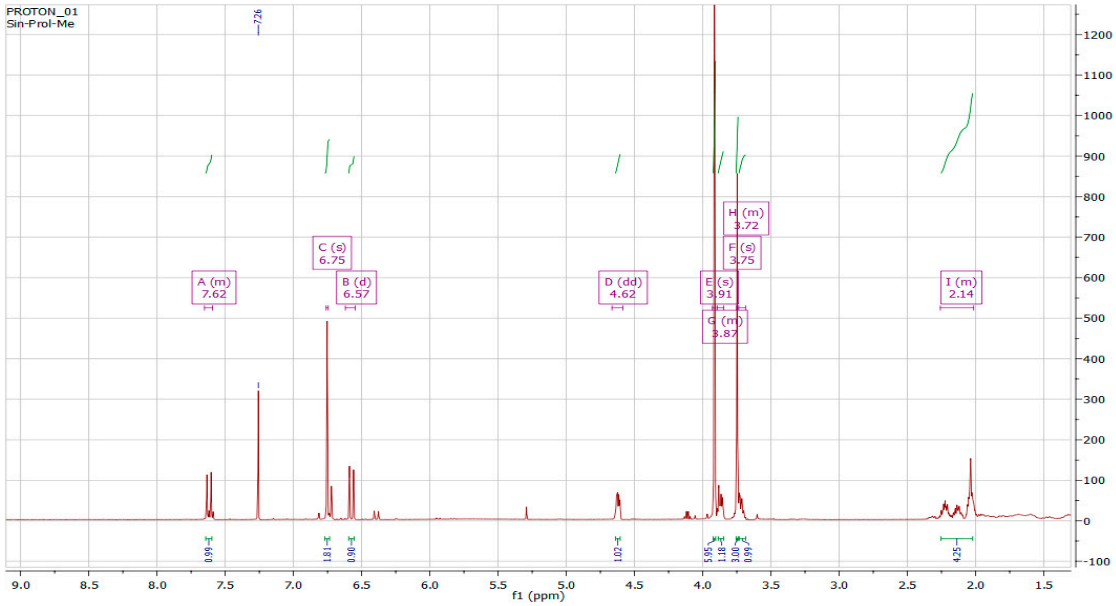

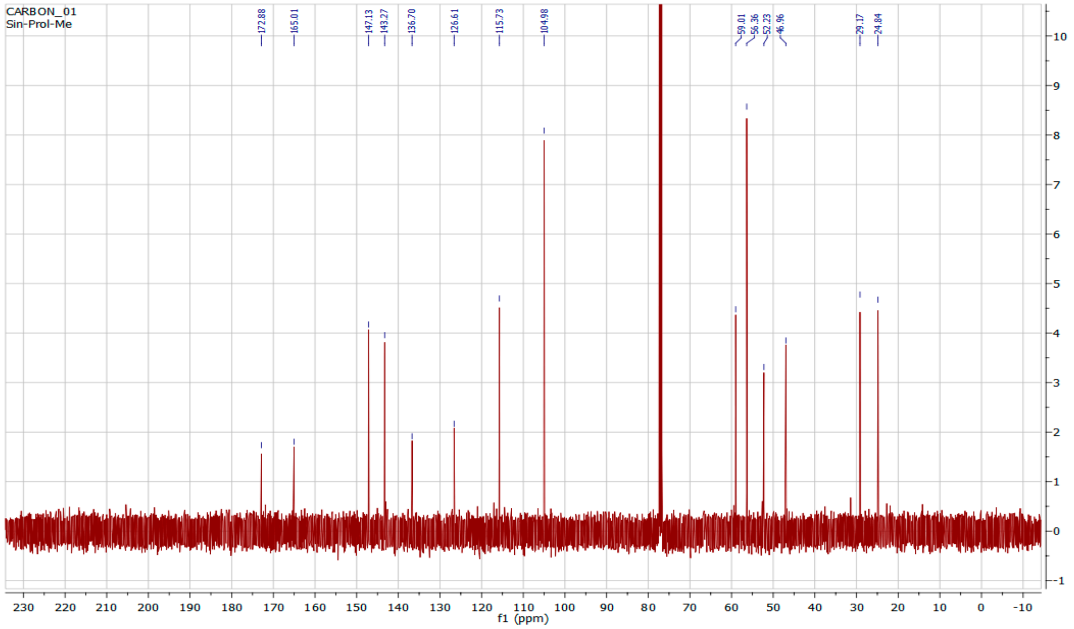

(*E*)-1-(3-(4-hydroxy-3,5-dimethoxyphenyl)acryloyl)pyrrolidine-2-carboxylic acid (**2b**)

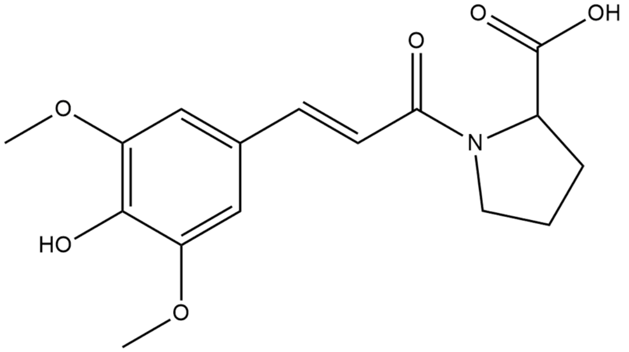

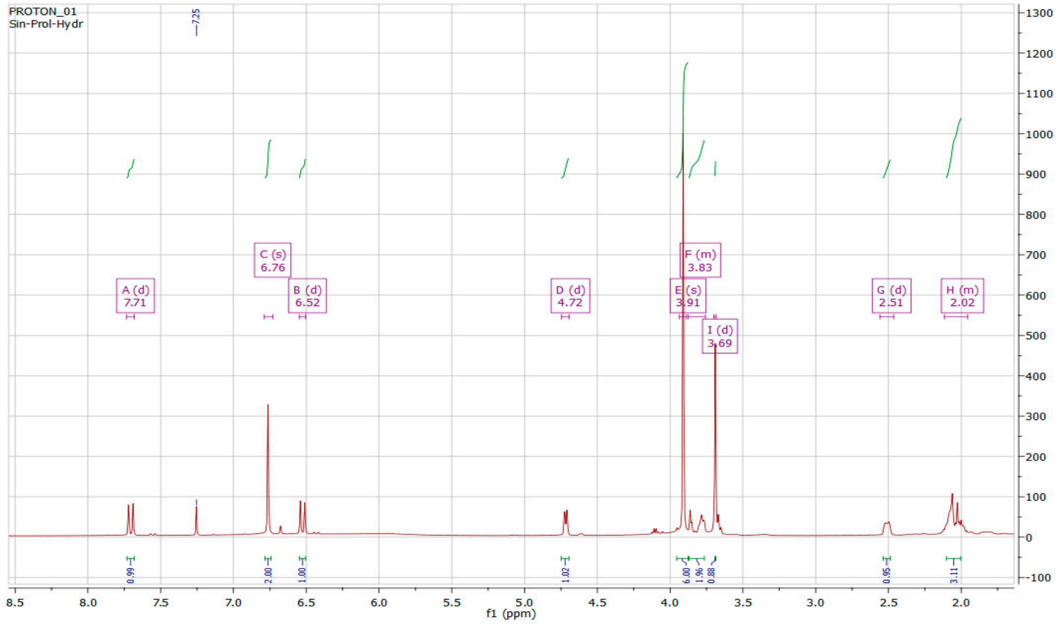

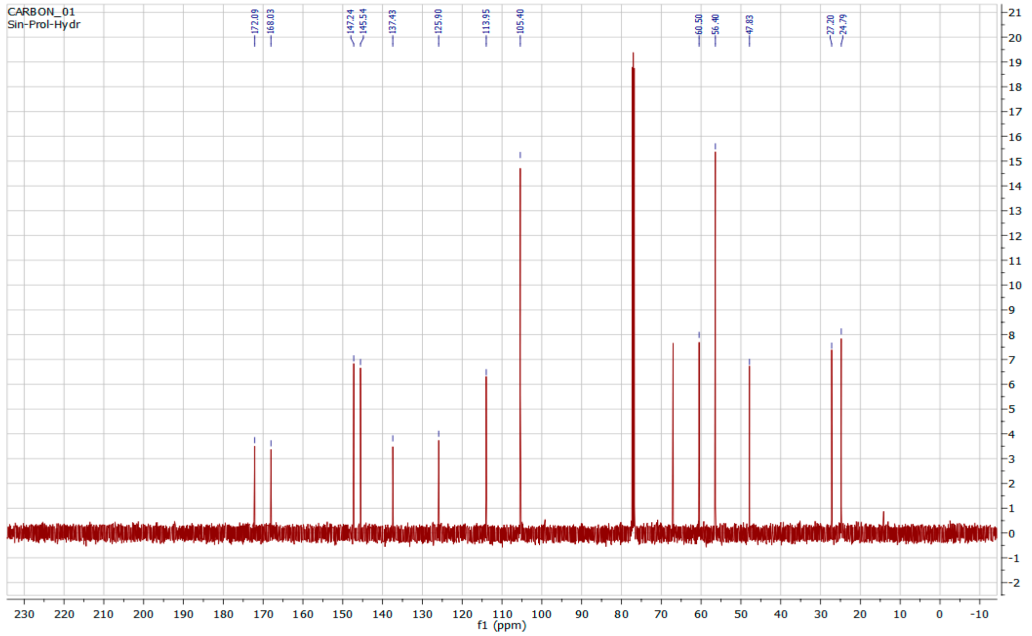

(E)-methyl 4-(1-(3-(4-hydroxy-3,5-dimethoxyphenyl)-acryloyl)pyrrolidine-2-carboxamido)-butanoate (**2c**)

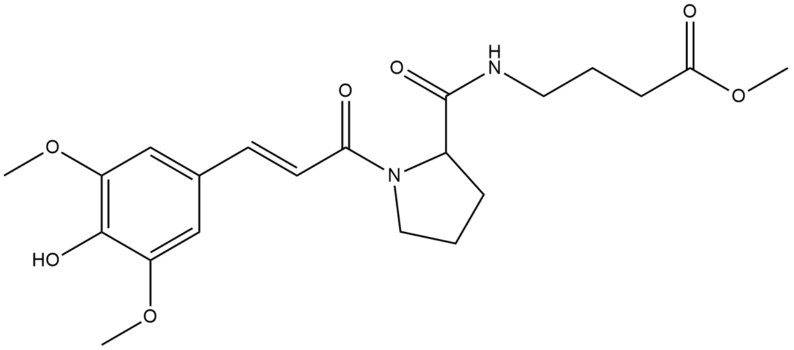

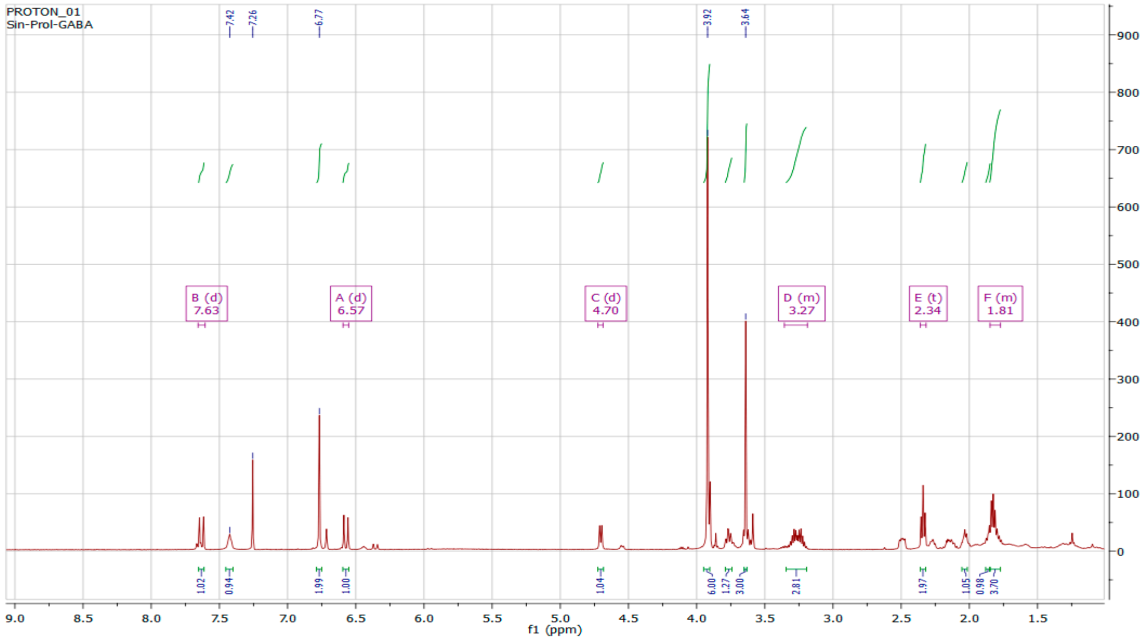

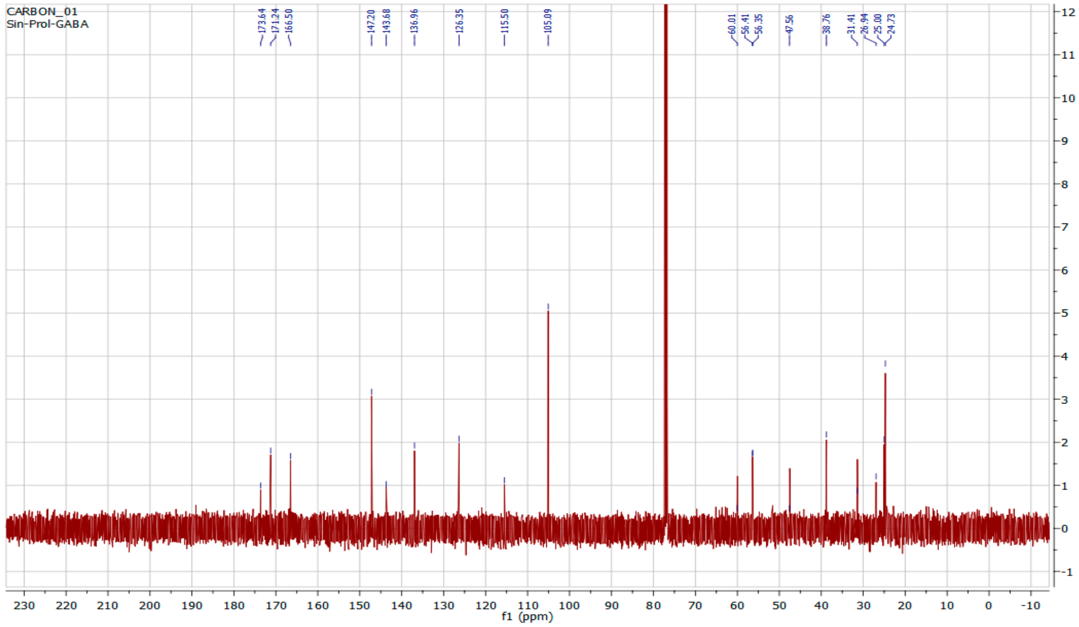

(*E*)-methyl 1-(3-(3,4-dimethoxyphenyl)acryloyl)pyrrolidine-2-carboxylate (**3a**)

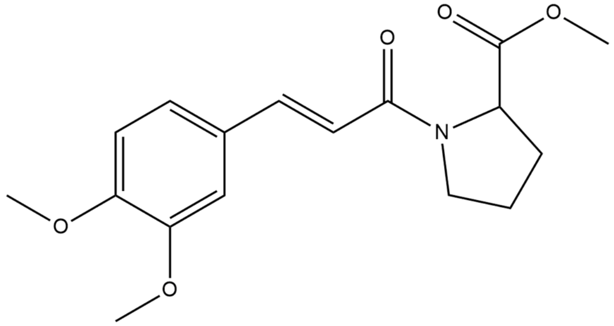

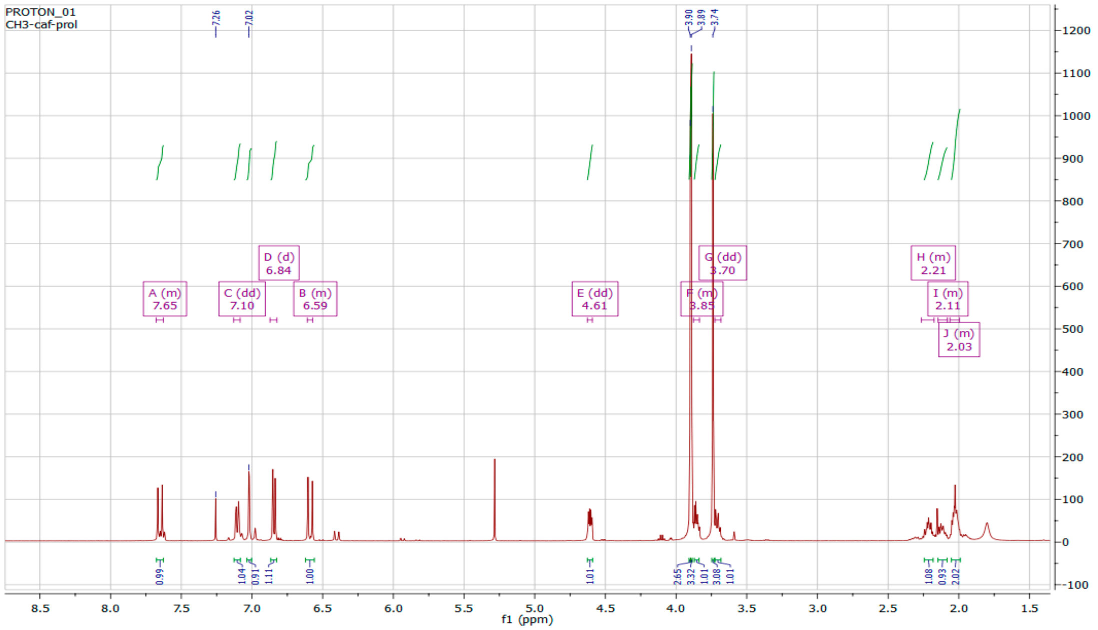

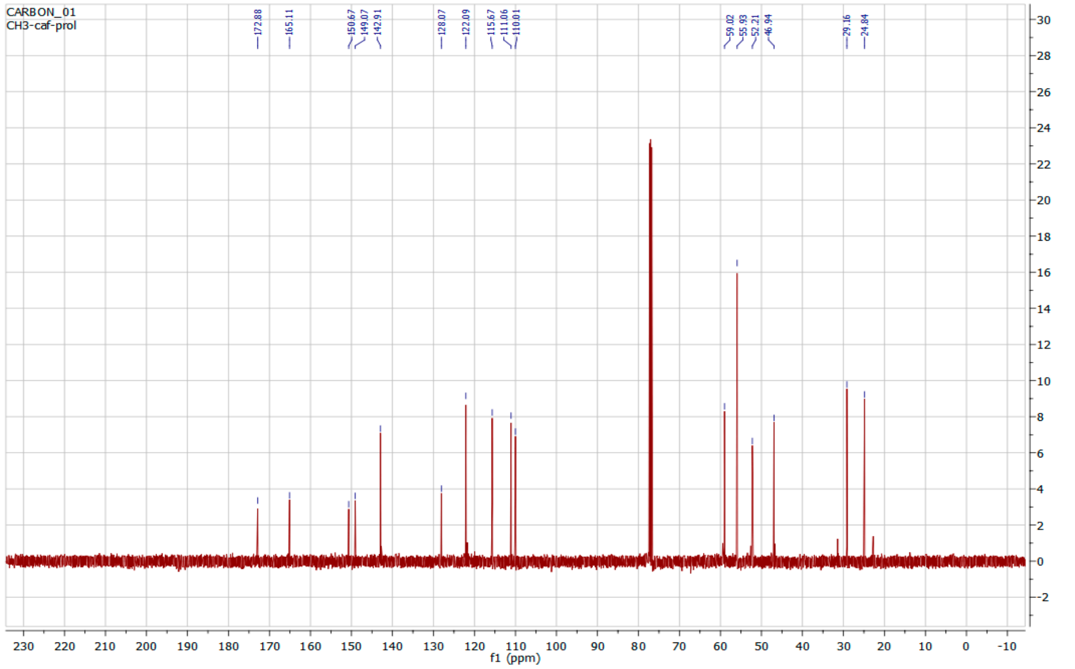

(*E*)-1-(3-(3,4-dimethoxyphenyl)acryloyl)pyrrolidine-2-carboxylic acid (**3b**)

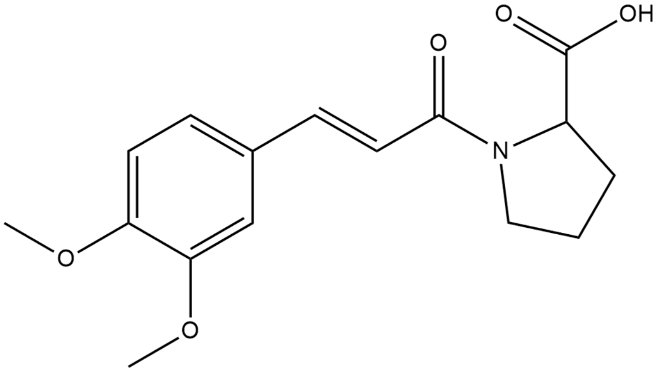

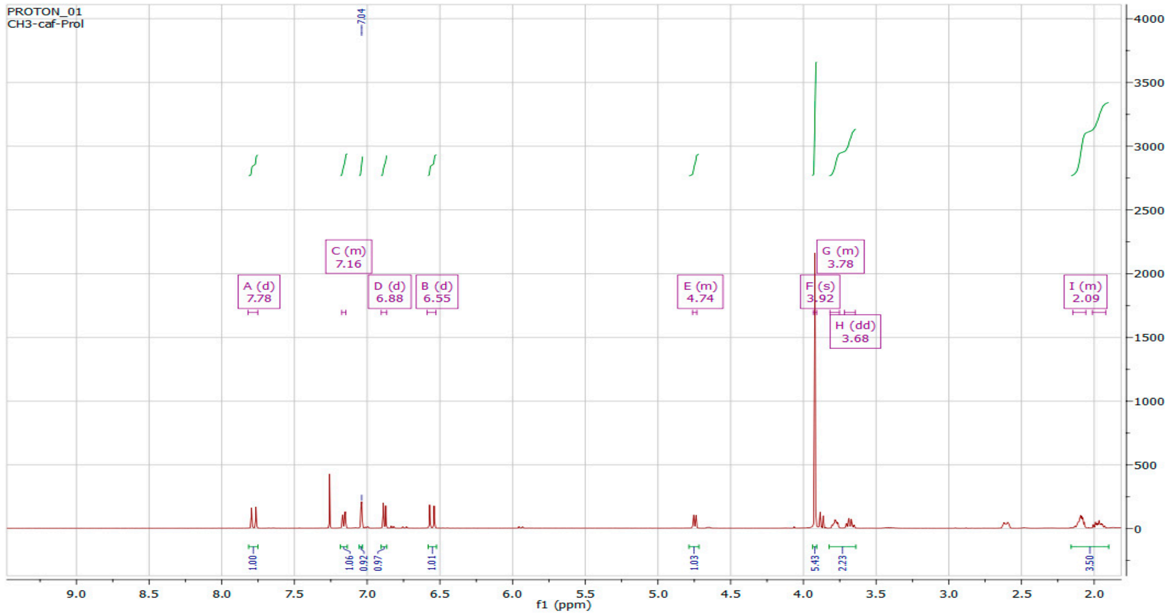

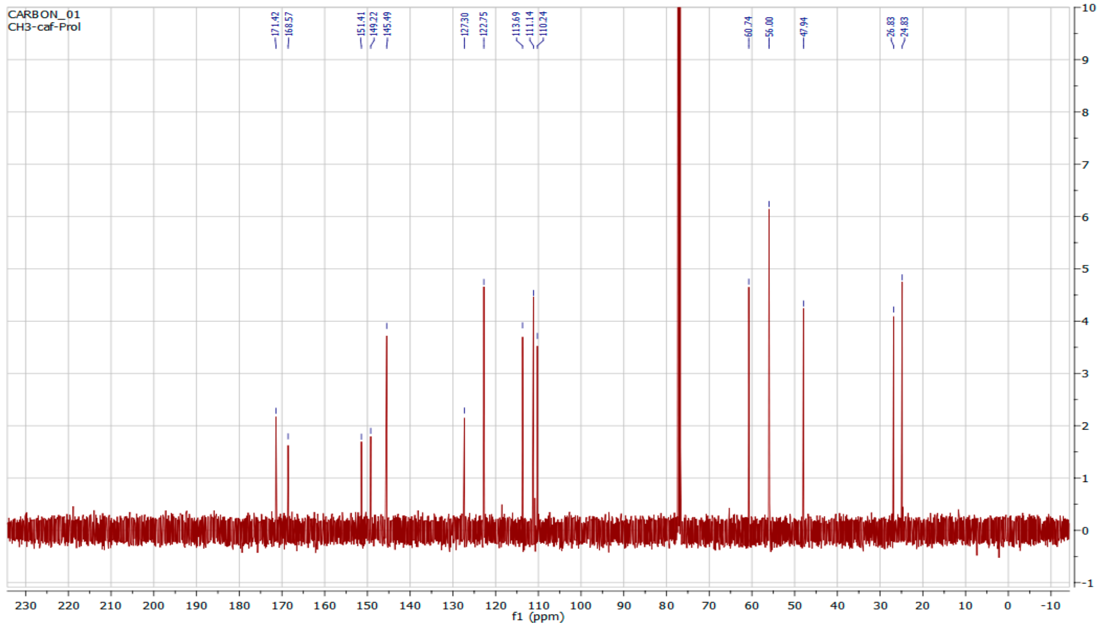

(*E*)-methyl 4-(1-(3-(3,4-dimethoxyphenyl)acryloyl)pyrrolidine-2-carboxamido)butanoate (**3c**)

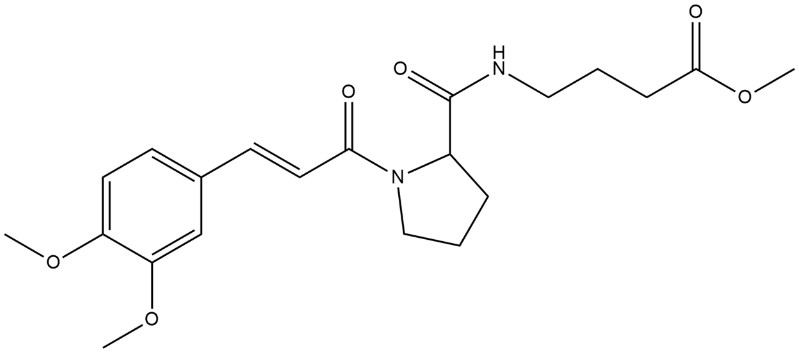

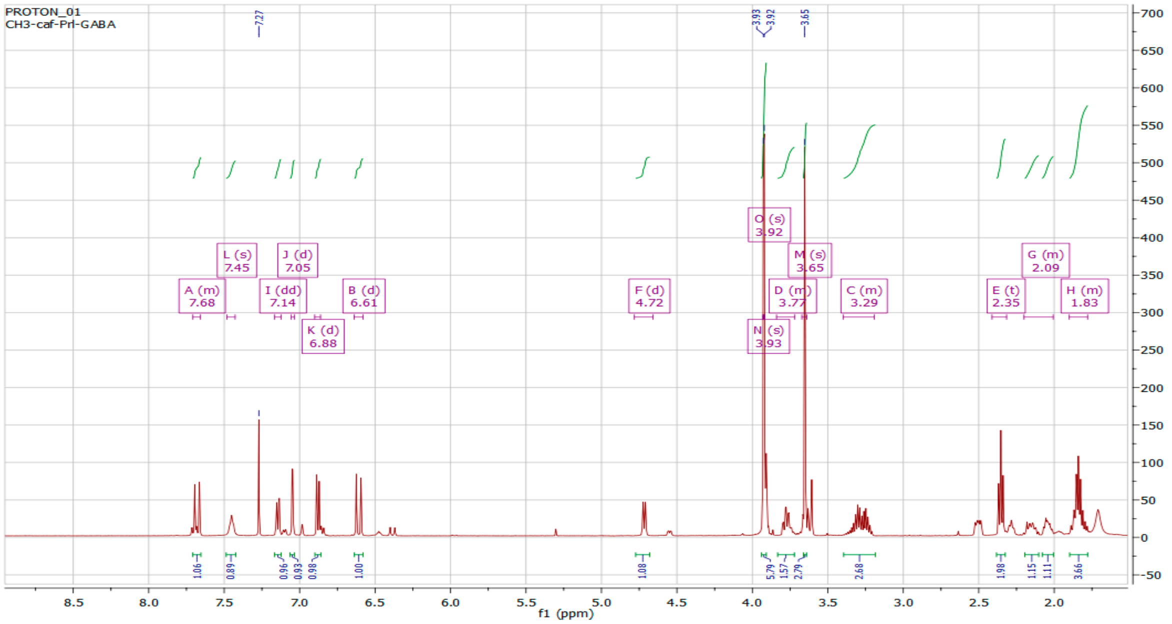

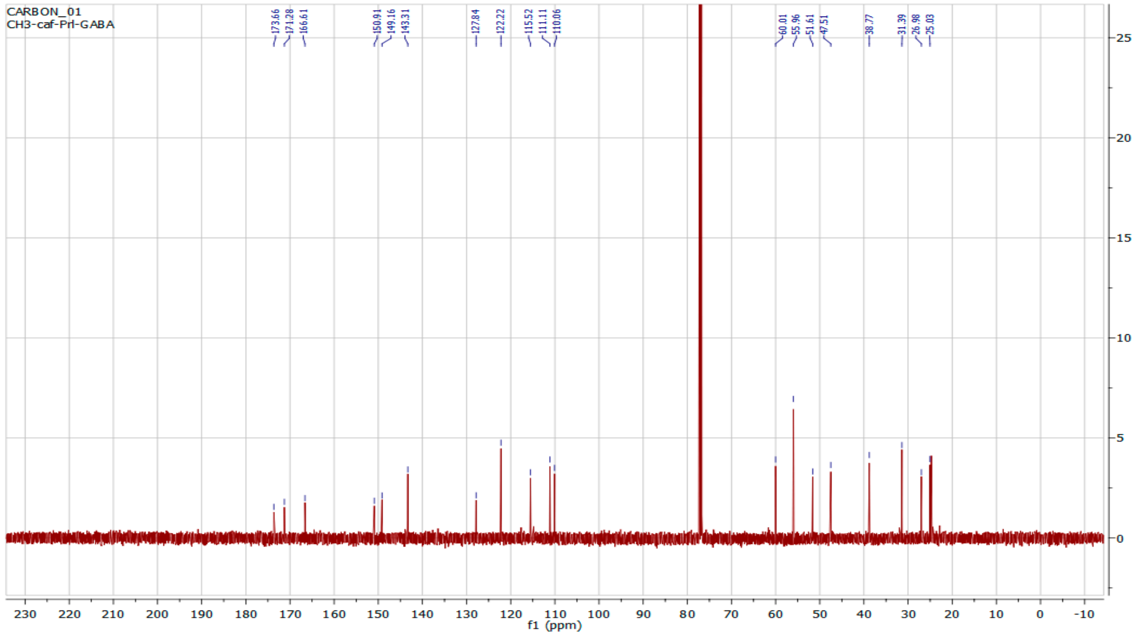

Methyl 1-cinnamoylpyrrolidine-2-carboxylate (**4a**)

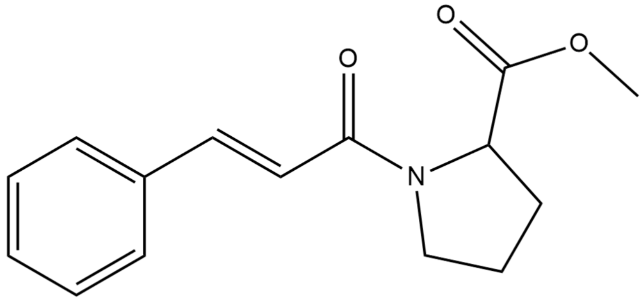

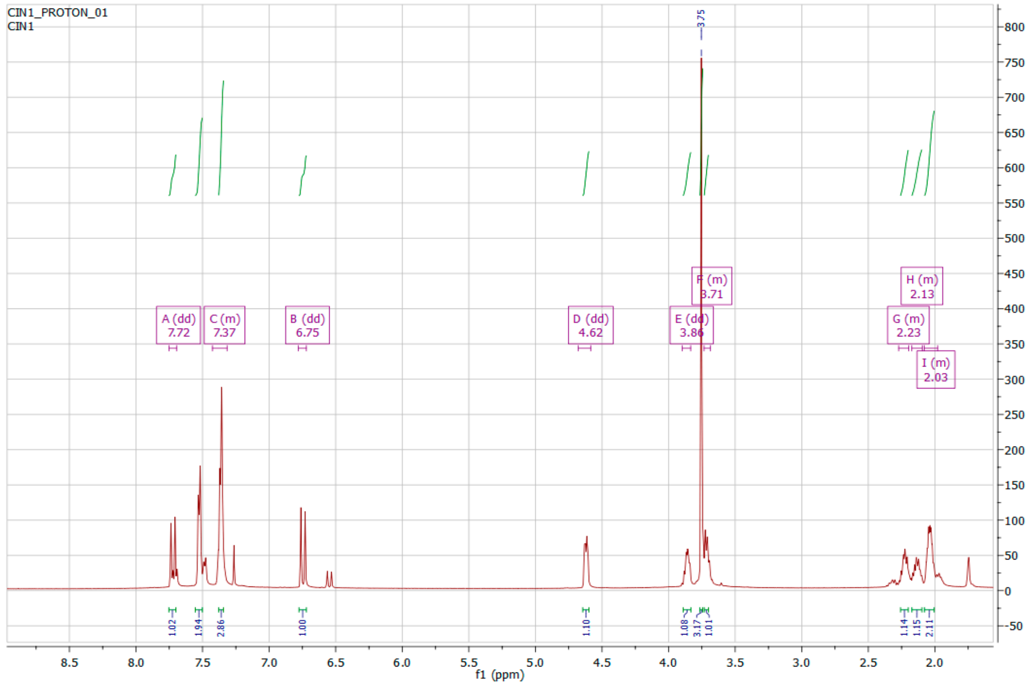

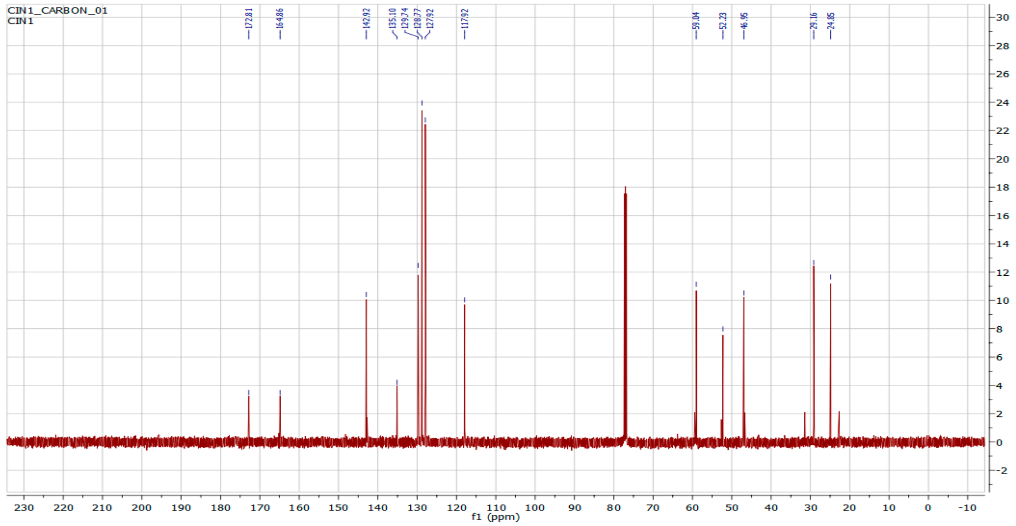

1-cinnamoylpyrrolidine-2-carboxylic acid (**4b**)

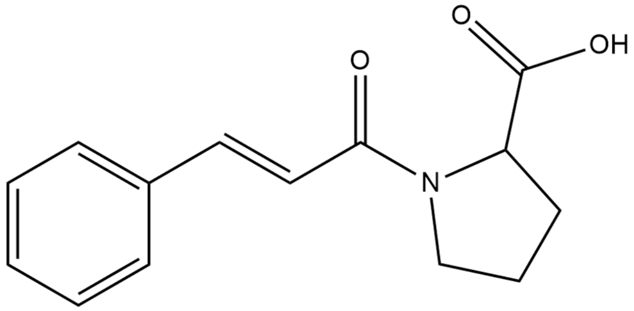

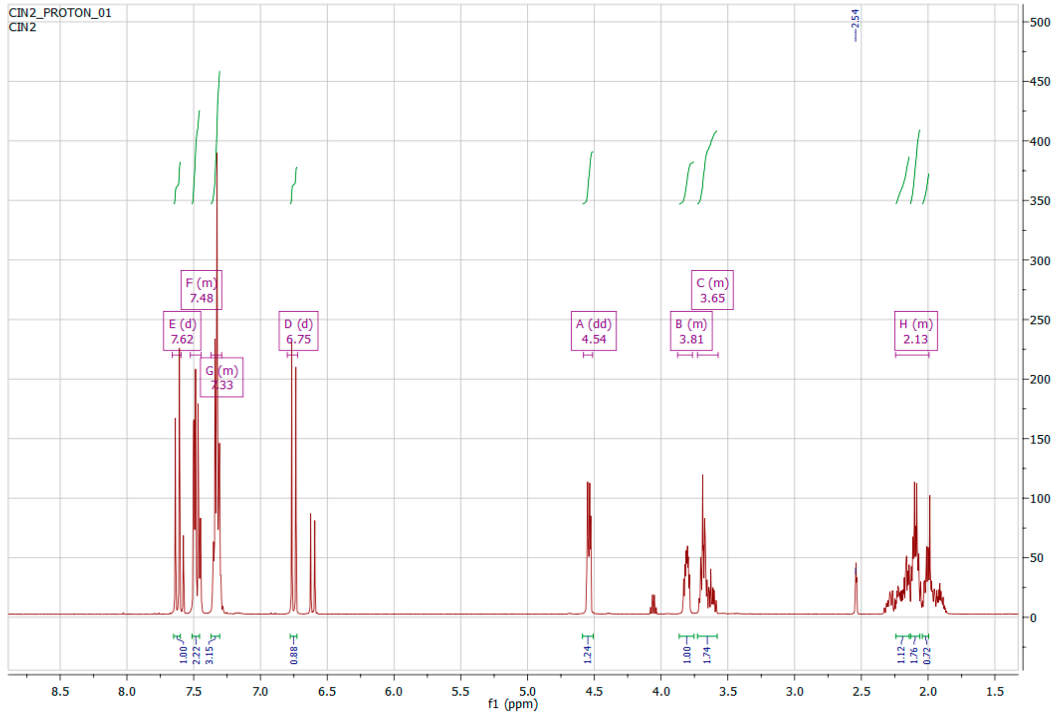

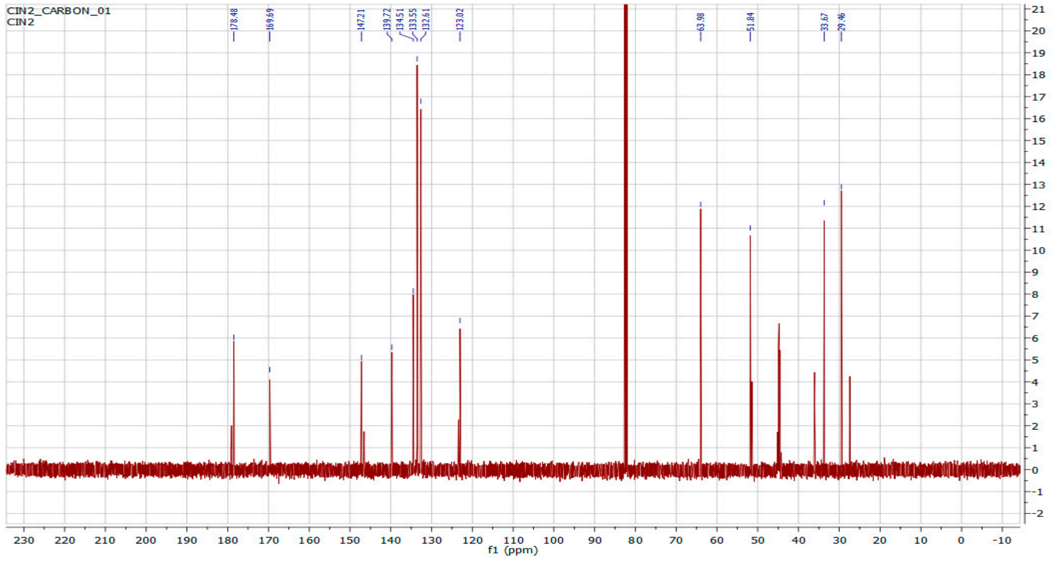

(E)-methyl 4-(1-cinnamoylpyrrolidine-2-carboxamido)butanoate (**4c**)

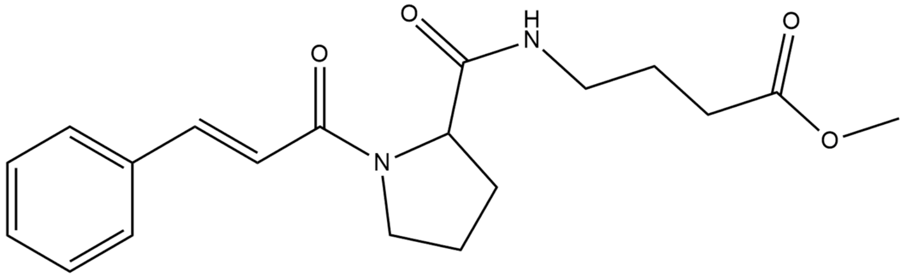

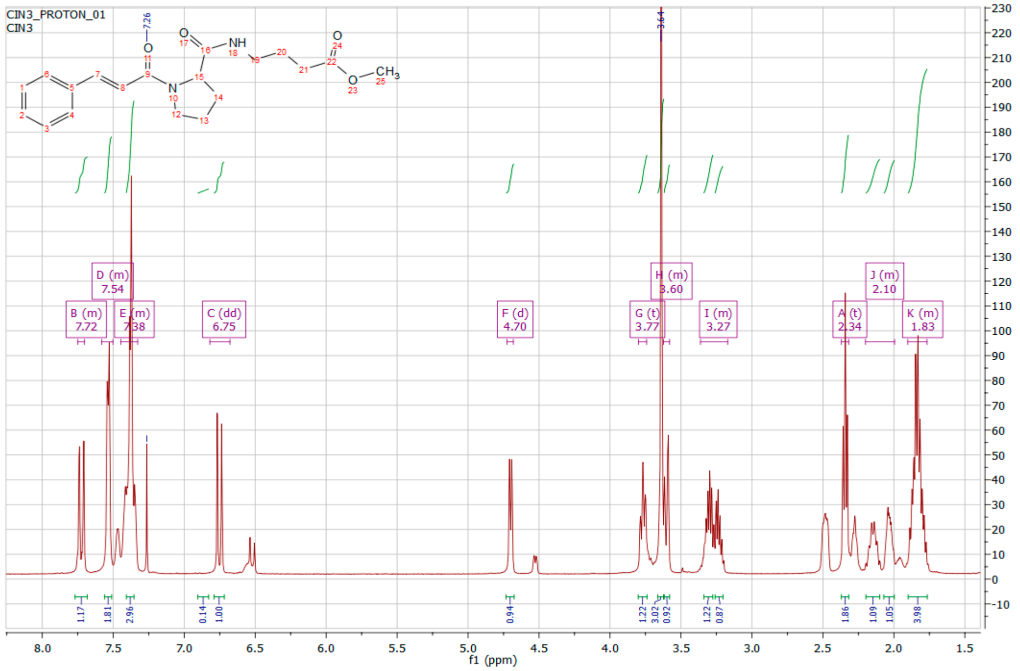

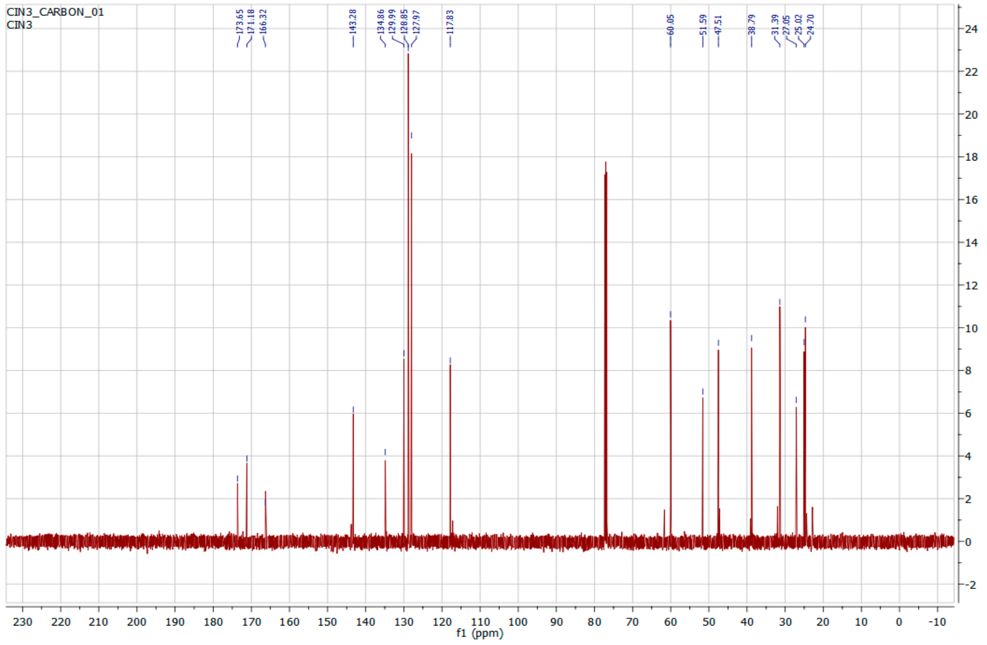

Methyl 1-(6-hydroxy-2,5,7,8-tetramethylchroman-2-carbonyl)pyrrolidine-2-carboxylate (**5a**)

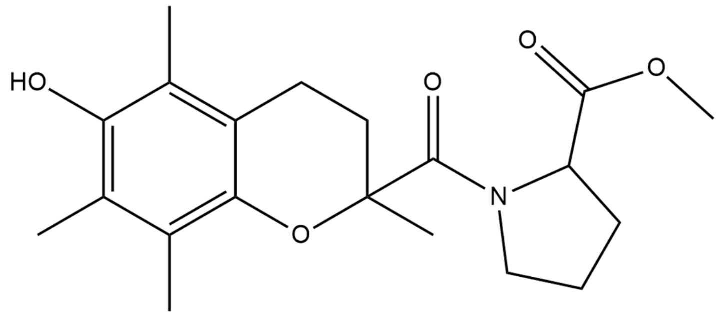

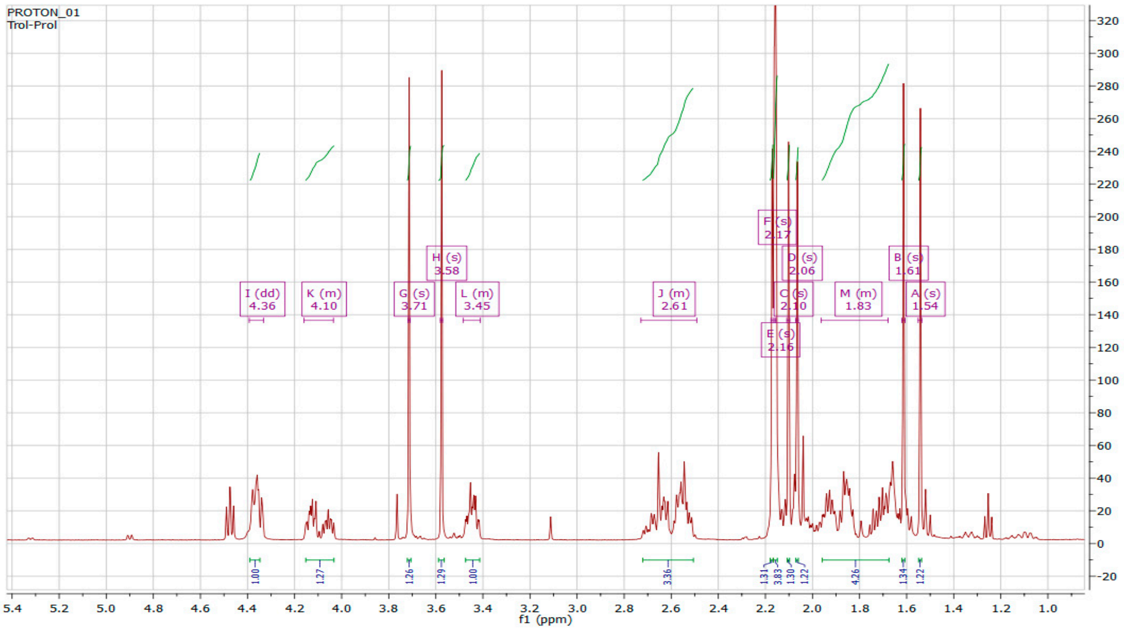

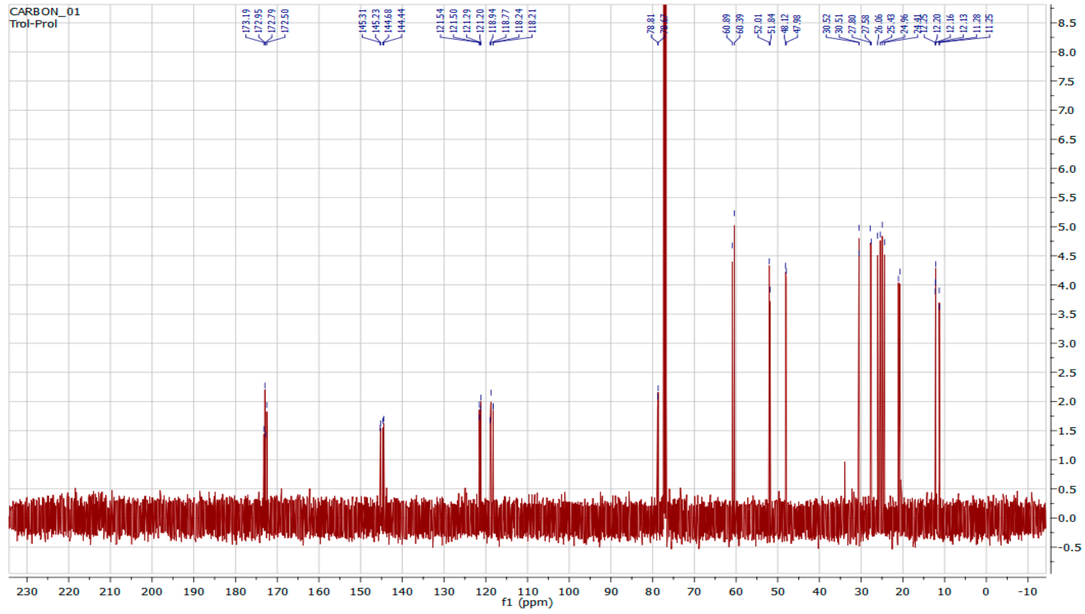

2-(6-hydroxy-2,5,7,8-tetramethylchroman-2-carbonyl)pyrrolidine-1-carboxylic acid (**5b**)

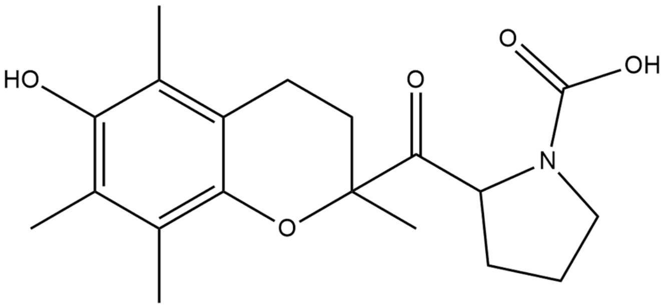

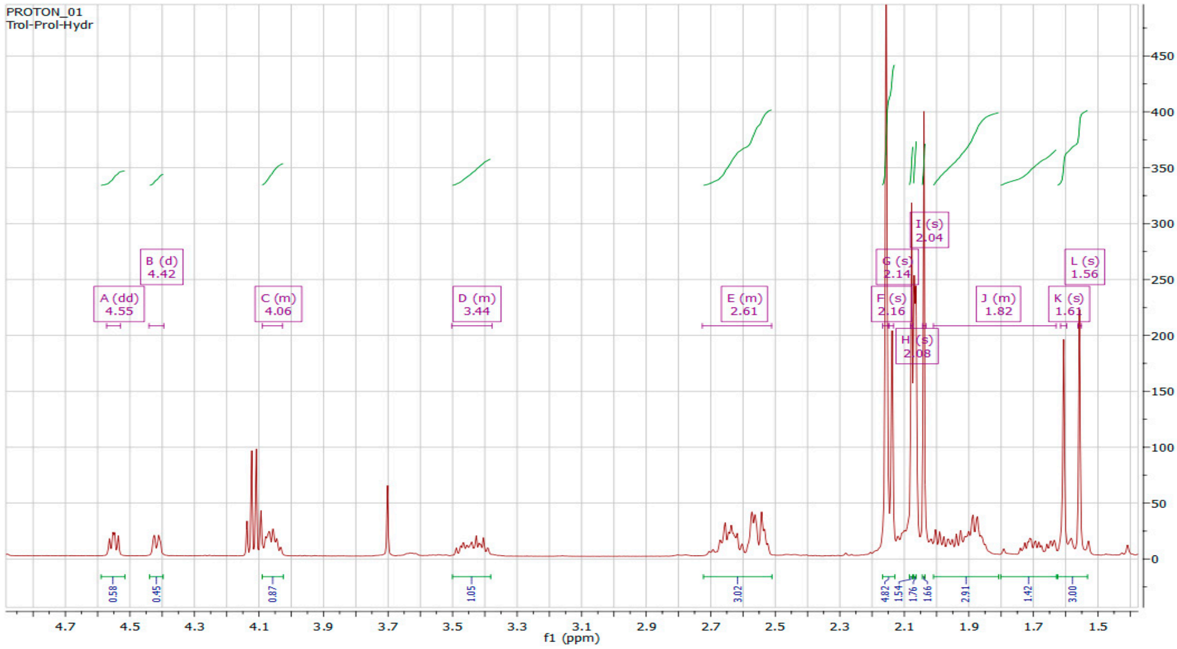

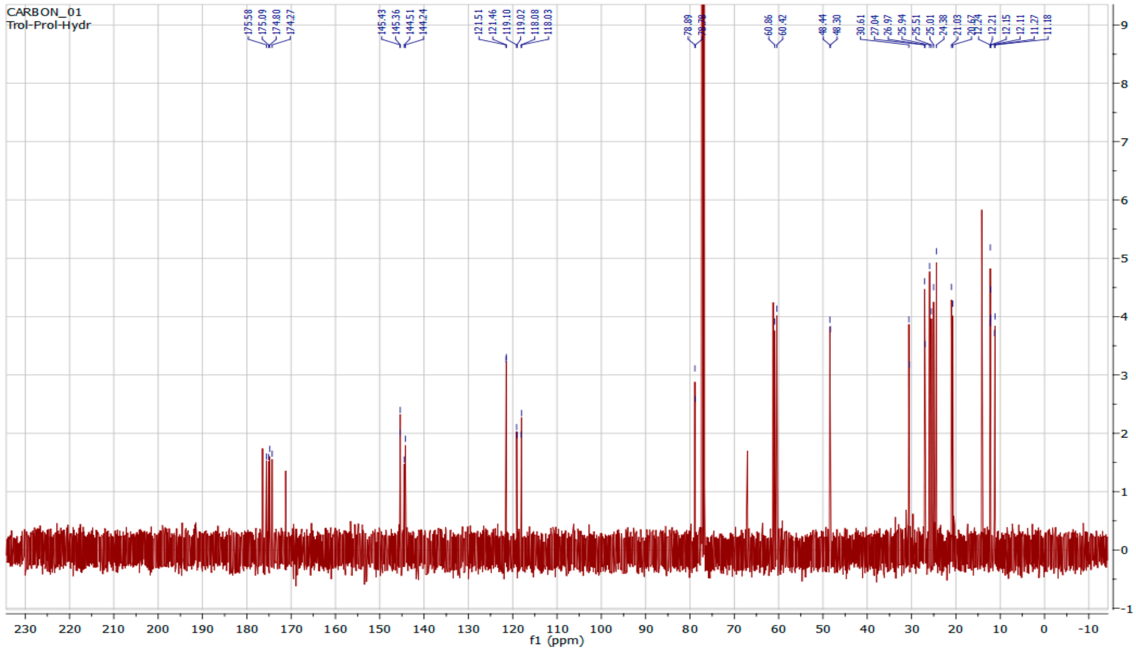

Methyl 4-(1-(6-hydroxy-2,5,7,8-tetramethylchroman-2-carbonyl)pyrrolidine-2-carboxamido)-butanoate

(**5c**)

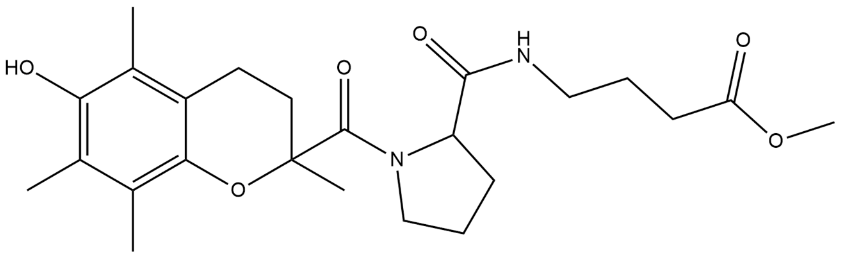

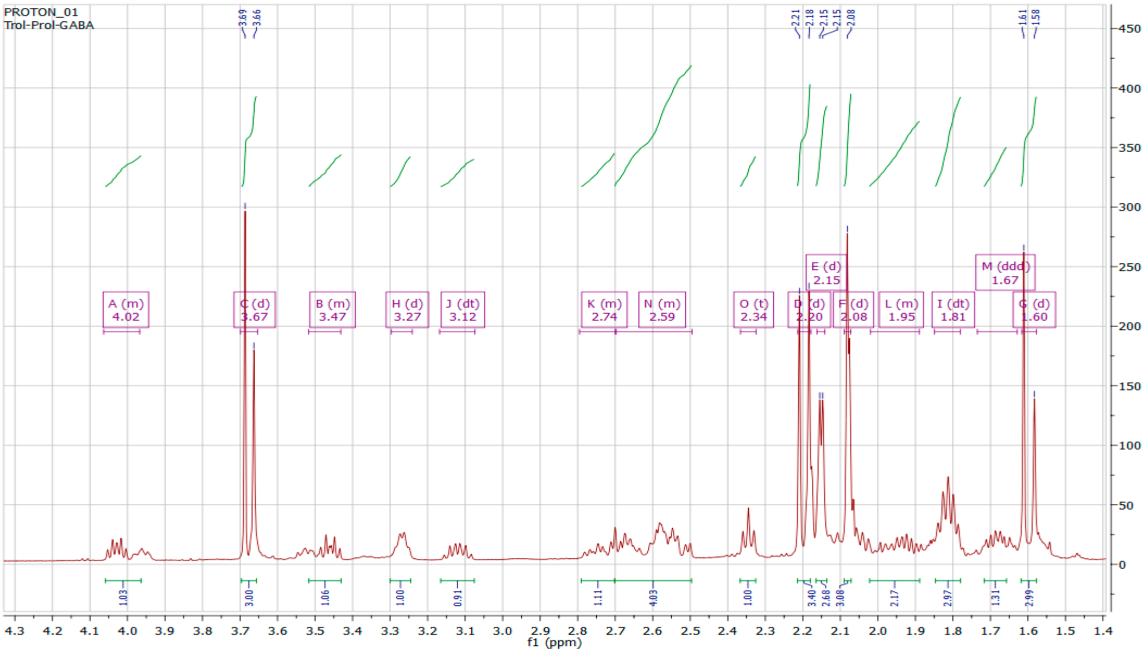

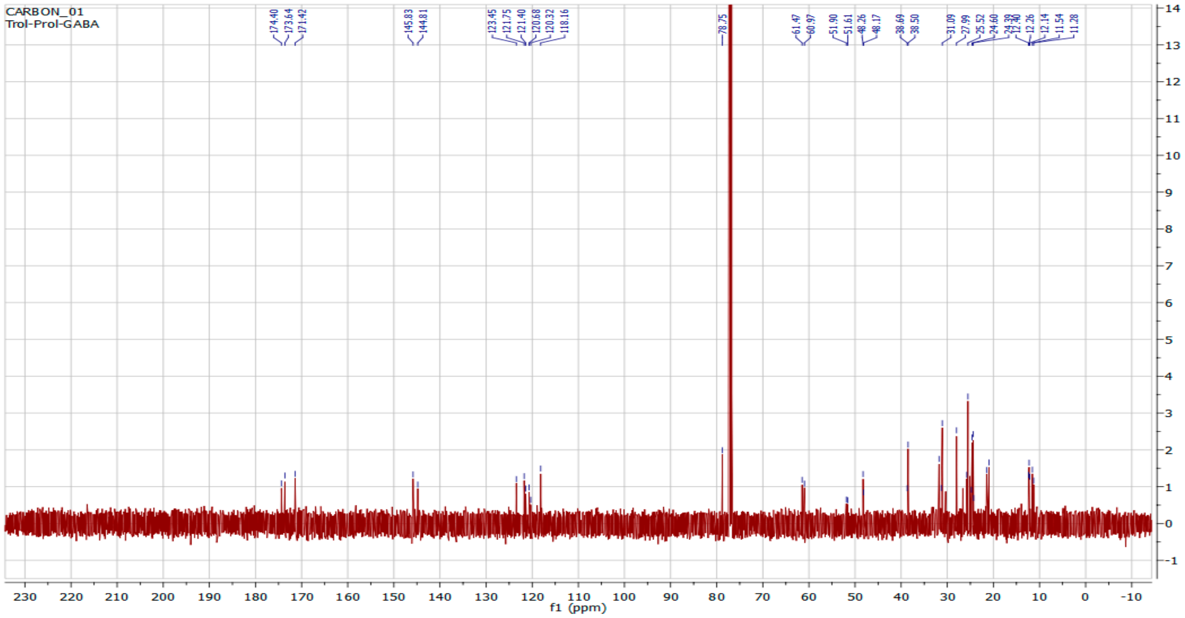

Supplement: Supplementary file 1 [file molecules-29-03763-s001.zip › molecules-3066755-supplementary.pdf]
